# Chromosome-level genome assembly for giant panda provides novel insights into Carnivora chromosome evolution

**DOI:** 10.1186/s13059-019-1889-7

**Published:** 2019-12-06

**Authors:** Huizhong Fan, Qi Wu, Fuwen Wei, Fengtang Yang, Bee Ling Ng, Yibo Hu

**Affiliations:** 1 CAS Key Laboratory of Animal Ecology and Conservation Biology, Institute of Zoology, Chinese Academy of Sciences, Beijing, China; 2 University of Chinese Academy of Sciences, Beijing, China; 3 Center for Excellence in Animal Evolution and Genetics, Chinese Academy of Sciences, Kunming, China; 4 Wellcome Sanger Institute, Wellcome Genome Campus, Hinxton, Cambridge, UK

**Keywords:** Giant panda, Chromosome-level genome, Chromosome evolution, Evolutionary breakpoint region, Carnivora

## Abstract

**Background:**

Chromosome evolution is an important driver of speciation and species evolution. Previous studies have detected chromosome rearrangement events among different Carnivora species using chromosome painting strategies. However, few of these studies have focused on chromosome evolution at a nucleotide resolution due to the limited availability of chromosome-level Carnivora genomes. Although the de novo genome assembly of the giant panda is available, current short read-based assemblies are limited to moderately sized scaffolds, making the study of chromosome evolution difficult.

**Results:**

Here, we present a chromosome-level giant panda draft genome with a total size of 2.29 Gb. Based on the giant panda genome and published chromosome-level dog and cat genomes, we conduct six large-scale pairwise synteny alignments and identify evolutionary breakpoint regions. Interestingly, gene functional enrichment analysis shows that for all of the three Carnivora genomes, some genes located in evolutionary breakpoint regions are significantly enriched in pathways or terms related to sensory perception of smell. In addition, we find that the sweet receptor gene *TAS1R2*, which has been proven to be a pseudogene in the cat genome, is located in an evolutionary breakpoint region of the giant panda, suggesting that interchromosomal rearrangement may play a role in the cat *TAS1R2* pseudogenization.

**Conclusions:**

We show that the combined strategies employed in this study can be used to generate efficient chromosome-level genome assemblies. Moreover, our comparative genomics analyses provide novel insights into Carnivora chromosome evolution, linking chromosome evolution to functional gene evolution.

## Background

Chromosome evolution is an important driver of speciation and species evolution. Carnivora species exhibit sharply contrasting karyotypes and provide excellent examples for studying chromosome evolution [1]. The karyotypes of the giant panda (2n = 42) and cat (2n = 38) are similar to that of the ancestral Carnivora which remains in ringtails today (2n = 38) [2], whereas the dog exhibits extensive chromosome reshuffling resulting in a complex karyotype (2n = 78). Some studies have established a series of comparative chromosome maps in different groups of Carnivora species using chromosome painting based on a fluorescent in situ hybridization (FISH) strategy [1–10]. Such studies have revealed interchromosomal and intrachromosomal rearrangements and led to the proposal of putative ancestral karyotypes for the entire Carnivora order [2]. However, these FISH-based cytogenetic methodologies do not provide a sufficient resolution to perform genome-scale synteny analysis or to accurately identify homologous synteny blocks (HSBs), fine-scale rearrangements, and evolutionary breakpoint regions (EBRs) [11]. In recent years, with the development of sequencing technology, an increasing number of Carnivora genomes have been sequenced. Based on these Carnivora genomes, some comparative genomic studies have been performed. For instance, comparisons among carnivore, omnivore, and herbivore genomes showed that carnivores are under strong selective pressure related to diet compared to the other two dietary groups [12]. However, few of these studies have focused on chromosome evolution at a nucleotide resolution due to the limited availability of high-quality chromosome-level genome assemblies for Carnivora species.

The giant panda (*Ailuropoda melanoleuca*), which is the epitome of a flagship species for wildlife conservation, provides a variety of ecosystem services that are valued locally and nationally [13]. Although the International Union for Conservation of Nature (IUCN) has downlisted the giant panda from “Endangered” to “Vulnerable,” it remains one of the most endangered mammals on Earth. According to the Fourth National Survey of Giant Pandas completed in 2012, the population size of giant pandas was estimated to be 1864 across 25,349 km^2^ of habitat [13]. The giant panda is also an ideal model of adaptive evolution [14]. As an obligate bamboo feeder, the giant panda has evolved unique morphological and physiological traits such as pseudothumbs and low energy metabolism rate to adapt to a low-nutrition and low-energy food [15]. Two versions of the giant panda genome have been assembled using next-generation sequencing (NGS) technology. The first version of the giant panda genome (scaffold N50 = 1.28 Mb) was used to investigate the genetic features underlying the unique biology of giant pandas [16]. The results showed that the giant panda exhibits a lower divergence rate than dogs but higher genetic variability than humans. The pseudogenization of *TAS1R1* may be related to the herbivorous diet of the giant panda [16]. Compared with the first version of the giant panda genome, the second version showed improved contiguousness (scaffold N50 = 9.95 Mb). This improved assembly was used to perform a comparative genomic analysis with the red panda genome [17]. Two limb development genes (*DYNC2H1* and *PCNT*), which have undergone adaptive convergence, may be important candidate genes for pseudothumb development in both pandas [17]. Although these two genomic assemblies can be used as reference genomes for population genomics and comparative genomics studies of giant pandas [17, 18], both of them were assembled based on short reads and may be fragmented and incomplete, making it difficult to study chromosome evolution at a nucleotide resolution.

Despite the rapid progress of sequencing technologies, it is difficult to obtain a high-quality chromosome-level genome with a low error rate until now. Recently, with the development of sequencing technologies, a combination of third-generation sequencing techniques [19, 20] and high-throughput chromatin conformation capture technique (Hi-C) [21] can produce high-quality genome assemblies, resulting in many chromosome-level draft genomes [22, 23]. However, most of the reads produced by third-generation sequencing technologies have a relatively high error rate of up to 10~15% [24]. Moreover, third-generation sequencing technology requires starting material consisting of hundreds of micrograms of high molecular weight DNA, and Hi-C technology requires large amounts of fresh samples. All of these factors limit the applications of these technologies in the field of conservation genomics due to the difficultly of obtaining large DNA samples for endangered animals. The alternative approach of 10X Genomics employs genome partitioning and barcoding to generate linked reads that span tens to hundreds of thousands of bases [25]. These linked reads can be used to scaffold the contigs [26]. 10X Genomics technology has been proven to be a cost-effective and robust strategy for producing high-quality genomes and has been successfully applied in the assembly of some plant and animal genomes [27, 28]. Additionally, previous studies have shown that the information provided by related reference genomes can be used to substantially improve the quality of a new assembly [29, 30], such as the Tasmanian devil genome assembly using the opossum genome [31], grass carp genome assembly using the zebrafish genome [32], and more recently giant and red panda genome assemblies using the dog genome [17].

In this study, based on previously published high-quality paired-end and mate-pair reads, 10X Genomics linked-reads , and the dog genome as an assisting reference, we first generated a high-quality draft genome of giant panda with a scaffold N50 of 23.47 Mb. Then, using the reads from flow-sorted chromosomes and the cat genome as an assisting reference, we arranged the scaffolds on the chromosomes and generated a chromosome-level giant panda genome with a total size of 2.29 Gb. We illustrated the utility of this new giant panda genome by exploring chromosome rearrangement events and detecting large-scale HSBs and EBRs among three Carnivora genomes. The findings provide novel insights into chromosome evolution and link chromosome rearrangements to the evolution of functional genes and trait adaptation.

## Results

### Genome assembly

Based on the previously published paired-end reads generated with Illumina sequencing platforms [17], the giant panda genome was assembled into contigs. Then, the primary contigs were scaffolded three times. First, using previously published mate-pair reads [16], the contigs were merged into scaffolds with N50 of 1.24 Mb. Then, a male giant panda was sequenced using 10X Genomics Chromium technology, and a total of 228 Gb linked-reads were generated (93.3-fold genome coverage). Using these 10X Genomics linked-reads, we extended the primary assembly to an assembly with a scaffold N50 of 18.04 Mb. Finally, based on the genome synteny results between giant panda and dog, we further extended the assembly and obtained a high-quality giant panda genome with a total size of 2.45 Gb and a scaffold N50 of 23.47 Mb (Additional file 1: Table S1). Within this newly assembled genome, approximately 93.6% of the genome was contained within 152 scaffolds larger than 1 Mb, with the largest spanning 81.38 Mb.

We compared the new giant panda genome with two previously published giant panda assemblies. The results showed that the new genome represented a substantial improvement, with the scaffold N50 being improved 18.3-fold and 2.4-fold over those of AilMel_1.0 [16] and ASM200744v1 [17], respectively (Table 1, Additional file 1: Figure S1). The total size of the new assembly was comparable with that of ASM200744v1 but greater than that of AilMel_1.0. The GC content of the new assembly was comparable with those of the two published assemblies (Table 1).

**Table 1:** Comparison of the new giant panda genome with previously published assemblies

	This study	ASM200744v1	AilMel_1.0
Total size of assembled scaffolds	2,445,001,150	2,428,263,693	2,299,509,015
Number of scaffolds	77,897	57,414	81,467
Scaffold N50	23,473,669	9,947,519	1,281,781
Scaffold L50	34	75	521
Longest scaffold	81,377,464	32,438,596	6,047,896
GC content	41.69%	41.69%	41.60%
Unresolved bases per 100 Kb	1937.47	1927.02	2356.86
Repeat region of assembly	41.05%	41.29%	34.7%
Number of gene models	22,284	23,371	22,154

### Genome synteny comparison among the giant panda, dog, and cat genomes

To investigate Carnivora genome conservation, we performed multiple genome alignment among the three genomes using Progressive Mauve software. The results showed that the total sizes of syntenic regions among the giant panda scaffolds and the dog and cat chromosomes were 2.29 Gb (93.7% of the genome), 2.27 Gb (93.1% of the genome), and 2.35 Gb (96.19% of the genome), respectively. The comparative results between the giant panda and dog genomes obtained in this study were similar to previously published results [16], which showed that the total sizes of syntenic regions between the giant panda and dog genomes were 2.22 Gb (96.7% of the genome) and 2.27 Gb (92.9% of the genome), respectively. The high level of synteny among the giant panda, dog, and cat genomes suggested that the sequences of these three genomes were largely conserved.

### Chromosome sequencing

We used a fibroblast cell line from a male giant panda to isolate and sequence each individual chromosome. The results showed that most of the giant panda chromosomes were individually sorted, but chromosome 9 cannot be resolved from chromosome X and chromosome 10 was mixed with chromosome 11. Each chromosome enriched by flow cytometry was sequenced to a depth between 66.5× and 267.6× with the Illumina X-Ten platform. We assigned the scaffolds from the above assembly to the giant panda chromosomes by mapping the flow-sorted chromosome paired-end sequence reads. A chromosome-level draft assembly was obtained, and the scaffold N50 of each chromosome ranged from 9.35 to 81.38 Mb (Additional file 1: Table S2).

### Chromosome construction and annotation

**Fig. 1:**
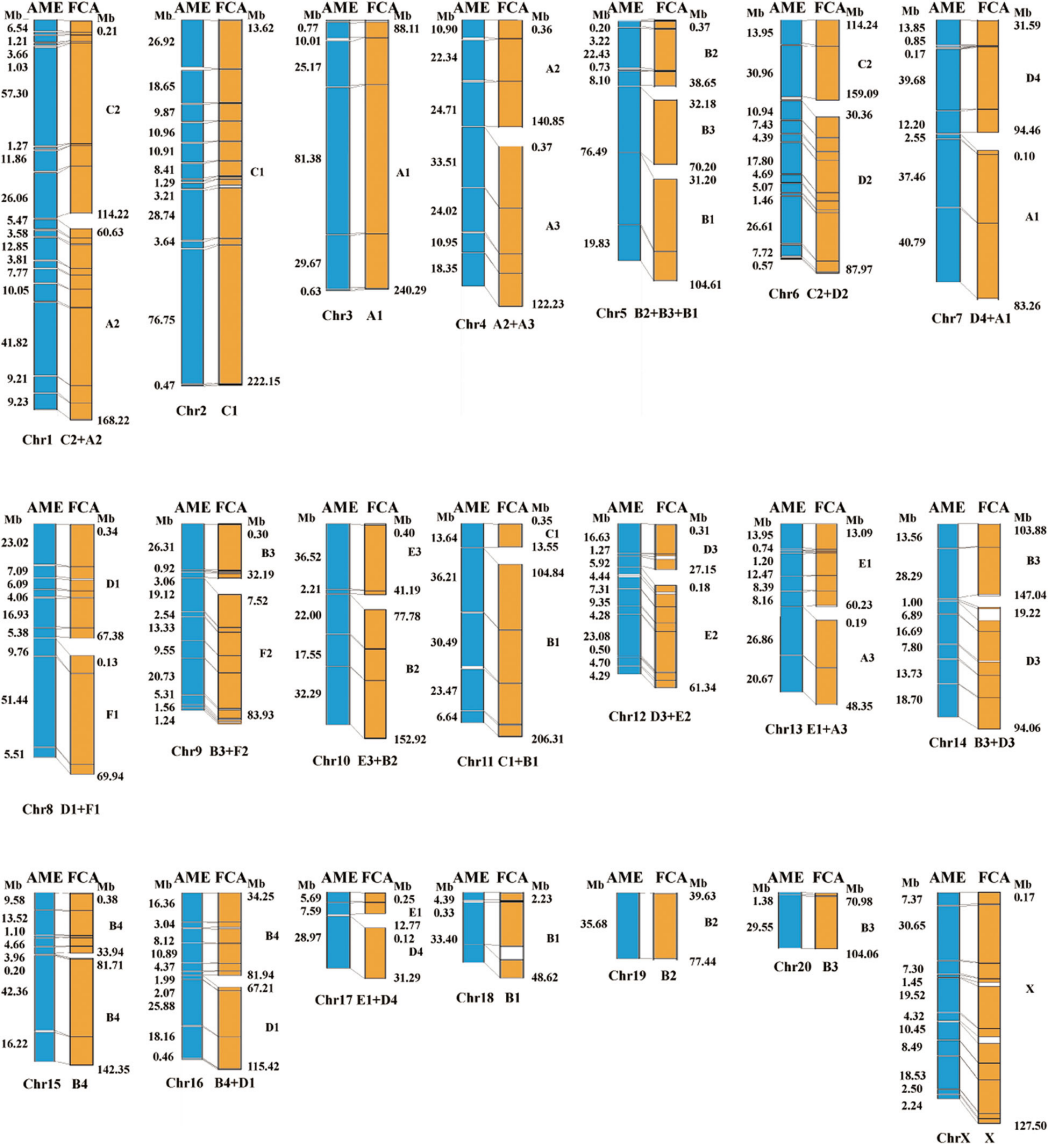
Construction of the chromosome-level genome of the giant panda through alignment with the cat genome. The assembled scaffolds of the giant panda (AME) genome (left, 2.29 Gb or 93.53% of the assembled genome) were aligned to the 19 cat (FCA) chromosomes. The blue and orange ideograms are the syntenic regions of the giant panda and cat genomes, respectively. The number on the left is the size of the giant panda scaffold, and the number on the right is start and end position of the aligned cat chromosomes

Because a genetic map of the giant panda was not available, the chromosome-level scaffolds could not be assigned to different linkage groups. To sort the chromosome-level scaffolds of the giant panda, we arranged the scaffolds on the same chromosome based on the synteny results between the giant panda and cat genome and previous cross-species chromosome painting results [1]. The results showed that a total of 164 scaffolds, covering 2.29 Gb (93.53%) of the assembled giant panda genome, were sorted based on directions of the cat chromosomes (Fig. 1). Moreover, the scaffolds assigned on two pairs of chromosomes, chromosomes 9 and X, and chromosomes 10 and 11, can be resolved to an individual chromosome using the evidence from the cat genome.

**Fig. 2:**
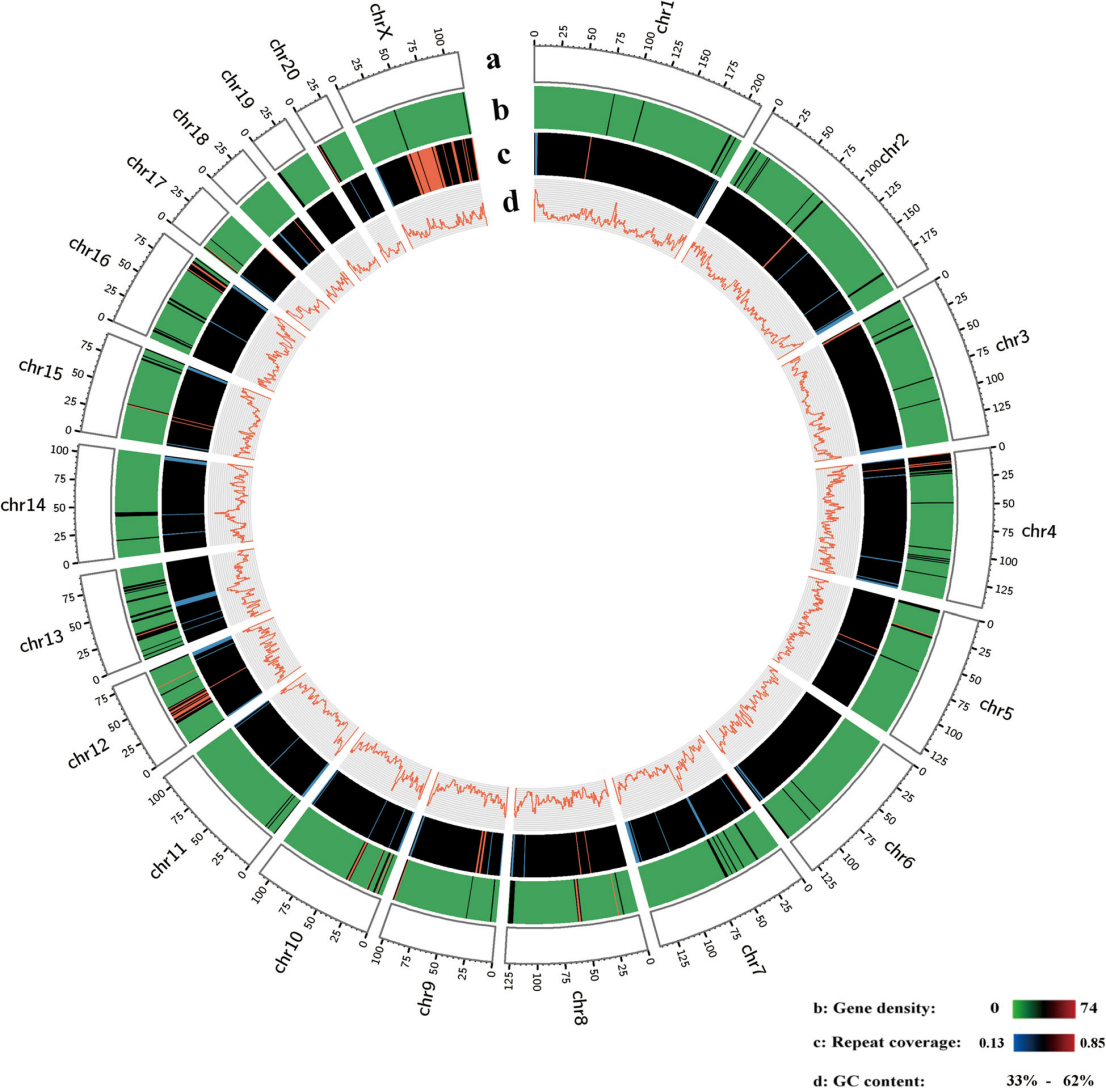
Characterization of the giant panda genome landscape. Circos plot of the multidimension topography of the giant panda genome, comprising 21 chromosomes that cover ~ 2.29 Gb of the genome assembly. The concentric circles, from outermost to innermost, represent **a** the ideogram of the 21 giant panda chromosomes (each tick mark is 5 Mb), **b** gene density (number of genes per Mb), **c** percentage of coverage of repeat sequence in 1 Mb windows, and **d** GC content. This figure was generated using Circos (http://circos.ca/)

Within this chromosome-level giant panda genome, a total of 933,158,675 repetitive sequences were identified, which were predominantly composed of LINEs and SINEs, constituting 40.81% of the giant panda genome. Using the Maker pipeline, we incorporated 40,418 transcripts assembled using giant panda RNA sequencing data and 78,431 protein sequences previously reported from giant panda, human, and dog genomes, and de novo predicted protein-coding genes. A total of 21,651 gene models were identified (Table 2), with the vast majority of gene predictions being supported by homology to known proteins or expressed transcripts. Based on this chromosome-level assembly and the corresponding gene annotation results, we calculated the GC content, gene density, and repetitive sequences of 21 chromosomes (Table 2) and mapped them to the whole giant panda genome (Fig. 2).

**Table 2:** The statistics and characteristics of the giant panda chromosomes

Chromosome	Chromosome size (Mb)	Anchored scaffold number	Anchored gene number	Percentage of repetitive sequences (%)	GC content (%)
Chr1	212.77	17	1481	38.05	39.16
Chr2	199.81	12	1772	39.82	40.34
Chr3	147.63	6	1073	38.72	39.92
Chr4	144.79	7	1731	38.25	42.48
Chr5	130.99	7	1176	41.13	39.98
Chr6	131.59	12	1060	39.53	41.69
Chr7	141.53	8	1033	37.81	39.88
Chr8	129.25	9	1466	39.82	41.59
Chr9	103.69	11	675	39.84	40.63
Chr10	110.58	5	1166	37.68	41.71
Chr11	110.51	5	825	39.06	39.87
Chr12	81.78	11	1536	38.12	45.88
Chr13	92.46	8	1471	37.06	45.13
Chr14	106.65	8	853	37.09	41.43
Chr15	91.61	8	764	39.04	41.21
Chr16	91.34	10	1421	39.06	41.88
Chr17	42.25	3	460	38.90	43.41
Chr18	38.12	3	236	39.59	38.94
Chr19	35.68	1	246	39.93	38.20
Chr20	30.94	2	332	40.72	38.32
ChrX	112.85	11	874	52.54	40.13
Sum	2286.84	164	21,651	–	–

### Chromosomal rearrangement among the giant panda, dog, and cat genomes

The chromosome-level de novo assembly of the giant panda and the published dog and cat genomes allowed us to detect chromosome rearrangements at a fine nucleotide resolution. Despite the overall strong collinearity observed among these three genomes, multiple chromosomal rearrangements were identified, including interchromosomal and intrachromosomal rearrangements. Because the relative orientations of the giant panda scaffolds were unknown, we focused on interchromosomal rearrangement events in the present study. When we aligned the dog and cat genomes to the giant panda chromosomes, a total of 59 and 16 chromosome fission events were identified, respectively. This was comparable to a previously published cytogenetic analysis [1] that identified 54 chromosome fission events between the giant panda and dog genomes, and 15 chromosome fission events between the giant panda and cat genomes (Additional file 1: Table S3).

### Identification and analysis of EBRs among the giant panda, dog, and cat genomes

To detect the potential EBRs, we determined large-scale HSBs among chromosome-level giant panda, cat, and dog genomes. Using SyMAP software [33], we aligned the cat and dog chromosomes to the giant panda chromosomes. The analyses identified a total of 97 large-scale HSBs between the giant panda and dog genomes (Additional file 1: Figure S2, Additional file 2: Table S4), a total of 38 large-scale HSBs between the giant panda and cat genomes (Additional file 1: Figure S3, Additional file 2: Table S5), and a total 85 large-scale HSBs between the dog and cat genomes (Additional file 1: Figure S4, Additional file 2: Table S6).

Based on the identified large-scale HSBs, we estimated the number and distribution of EBRs among these three genomes. In this study, we focused on the EBRs caused by chromosome fission events. The results showed that alignment of the dog and cat genomes to the giant panda chromosomes revealed a total of 58 and 15 EBRs, respectively, 14 of which presented overlapped regions in the giant panda genome (Additional file 1: Table S7). Alignment of the giant panda and dog genomes to the cat chromosomes revealed a total of 17 and 48 EBRs, respectively, 10 of which exhibited overlapping regions in the cat genome (Additional file 1: Table S8). Alignment of the giant panda and cat genomes to the dog chromosomes revealed a total of 36 and 28 EBRs, respectively, 27 of which exhibited overlapping regions in the dog genome (Additional file 1: Table S9). By taking the union of these EBRs in each genome, a total of 59, 37, and 55 EBRs, covering 18.91 Mb, 24.21 Mb, and 38.18 Mb, were identified in the giant panda, dog, and cat genomes, respectively (Additional file 1: Table S10). The length of EBRs in the giant panda genome ranged from 8463 bp to 2.49 Mb, with an average length of 320.45 Kb; the length of EBRs in the cat genome ranged from 53.95 Kb to 6.09 Mb, with an average size of 694.20 Kb; and the length of EBRs in the dog genome ranged from 86.30 Kb to 2.48 Mb, with an average size of 654.58 Kb (Additional file 1: Table S11). In addition, we found that the numbers of EBRs in the giant panda (AME) and cat (FCA) chromosomes varied (from 0 in AME18 and AME19 to 8 in AME2; from 1 in FCA_E1 to 10 in FCA_C1)  (Additional file 1: Table S10).

### Genome features of EBRs in the three Carnivora genomes

To investigate the genome features of EBRs in three Carnivora genomes, we first performed repeat element annotation analysis and found that the EBRs in the giant panda, cat, and dog genomes were mainly enriched for LINE-L1 elements (Additional file 1: Table S12). Moreover, we compared the gene density, GC content, and repeat element in the EBRs with those in the whole genome. The results showed that for the giant panda and dog genomes, the values for these three genome features were significantly higher in the EBRs than those in the whole genome (Additional file 1: Table S13 and Additional file 1: Table S14). However, for the cat EBRs and genome, no significant difference was detected for the three genome features (Additional file 1: Table S15).

### Functional categories of genes located in EBRs

**Fig. 3:**
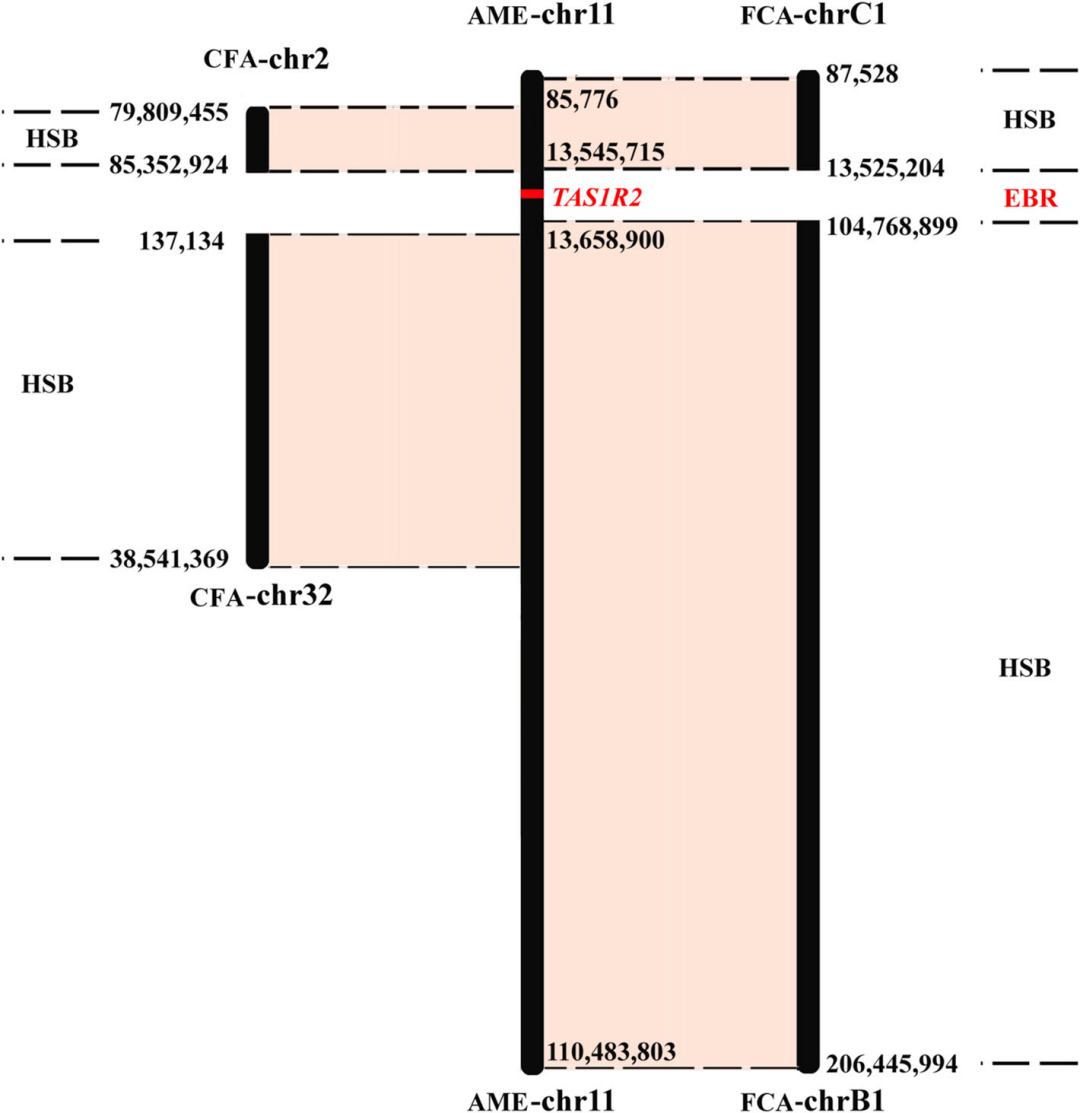
One case of EBR in the giant panda (AME) genome which included the functional gene *TAS1R2.* In contrast, this corresponds to an interchromosomal fission in dog (CFA) and cat (FCA) genomes. *TAS1R2* has a functional homologous gene in the dog genome, but its homologous gene in the cat genome has been proven to be pseudogenized

Using the BioMart data management system of the Ensembl genome browser, we identified a total of 342, 549, and 480 genes in the EBRs of the giant panda, dog, and cat genomes, respectively. We further performed homology analysis of the EBR genes among the three genomes to identify species-specific genes. The results revealed a total of 17, 168, and 132 species-specific genes in the EBRs of the giant panda, dog, and cat genomes. Interestingly, we also found that the intact sweet receptor gene *TAS1R2*, which is located in an EBR of giant panda, has a functional homologous gene in the dog genome, but the corresponding homologous gene in the cat genome has been proven to be a pseudogene (Fig. 3) [34–36]. The pseudogenization of *TAS1R2* may make cat insensitive to sweet-tasting compounds compared to dog’s normal sweetness taste [34–36].

To determine whether there are functional categories that are preferentially overrepresented in EBRs, we performed GO and KEGG pathway functional enrichment for the genes located in the EBRs of the giant panda, dog, and cat genomes, respectively. The results showed that some genes located in giant panda, dog, and cat EBRs were significantly enriched in the sensory perception of olfaction (Additional file 2: Table S16-S18).

## Discussion

A chromosome-level reference genome assembly is a valuable resource for conservation evolutionary biology [37] and conservation genomics studies of endangered species [38]. Such an assembly is essential for studies on chromosome evolution and lineage-specific adaptation. However, the assembly of a high-quality chromosome-level genome remains a difficult goal to realize. In this study, using a combination of flow-sorting, 10X Genomics, and reference-assisted assembly strategies, we successfully constructed a chromosome-level giant panda genome. First, using 10X Genomics linked-reads , we improved the scaffold N50 of the primary giant panda genome from 1.24 to 18.04 Mb. This assembly contained scaffolds that were nearly 15-fold longer than those obtained from paired-end and mate-pair sequencing reads. This was a large step toward improving the de novo genome assembly. Then, using the synteny information between the giant panda and dog genomes, we further improved the scaffold N50 from 18.04 to 23.47 Mb (Additional file 1: Table S1). Finally, using flow-sorting sequencing reads for each chromosome and cross-species chromosome painting results between the giant panda and cat, we successfully assigned over 93% of the scaffolds to giant panda chromosomes. The anchoring rate was comparable to that of genomes assembled using high-density genetic mapping or Hi-C technology. In this study, only a small DNA sample was required, which is an important prerequisite in the field of conservation genomics because it is difficult to obtain large DNA samples for endangered animals. Overall, the assembly strategy used in this study is more suitable for the construction of chromosome-level reference genomes for endangered species.

The role of chromosome evolution in speciation and adaptation has long been of interest to evolutionary biologists. Our chromosome-level draft assembly of the giant panda genome combined with published chromosome-level dog and cat genomes provides opportunities to identify novel chromosome rearrangement events among these three Carnivora genomes that previous cytogenetics approaches could not identify. In this study, due to the unknown orientations of the giant panda scaffolds, we focused on chromosome fission events among the giant panda, dog, and cat genomes. When the dog and cat genomes were mapped to the giant panda chromosomes, we identified a total of 59 and 16 chromosome fission events. We compared these chromosome fission events with those identified in a previously published cytogenetic analysis [1]. The results showed that all of 15 chromosome fission events between giant panda and cat detected via cytogenetic analysis were also identified in our study, whereas one novel chromosome fission event between giant panda and cat was identified in our study but not in the cytogenetic analysis. Six novel chromosome fission events between giant panda and dog were identified in our study but not in the cytogenetic analysis, whereas one chromosome fission event was identified in the cytogenetic analysis but not in our study (Additional file 1: Table S3).

In this study, by performing synteny analysis among three chromosome-level Carnivora genomes, a total of 59, 37, and 55 EBRs were identified in the giant panda, dog, and cat genomes. The relatively small number of EBRs in dog may be related to the fact that the dog presents the highest chromosome number among the Carnivora species. Previous studies revealed that EBRs are associated with several genomic features, such as high GC sequences [39], gene-rich regions [40], chromosome fragile sites [41], and elevated frequencies of segmental duplications and repeat elements [42]. In this study, the results showed that significant increases in gene density, GC content, and repeat elements were observed in the EBRs compared with the whole genome for the giant panda and dog, respectively, but no significant differences were detected between the cat EBRs and whole genome because the karyotype of the cat is closer to that of ancestral carnivore karyotype as compared to those of the dog and giant panda [3, 43].

It has been demonstrated that EBRs are evolutionarily unstable regions due to a high frequency of repeat elements [42]. Elsik et al. [44] identified 124 cattle-specific EBRs and found that the density of LINE-L1 and LINE-RTE was significantly higher in EBRs than those in the whole genome. Groenen et al. [45] detected 192 pig-specific EBRs and found that pig-specific EBRs were enriched for LTR-ERV1 and satellite repeats. In this study, by analyzing the repeat contents of giant panda, dog, and cat EBRs, significantly higher proportions of LINE-L1 elements were identified, indicating that LINE-L1 may contribute to the chromosome evolution.

Chromosome breakage during evolution is nonrandom. The EBRs appear to be hotspots of evolutionary activity where novel genes may be created. These genes may contribute to the adaptation of species [46]. In this study, we performed homology analysis of EBR genes and identified a total of 17, 168, and 132 species-specific genes in the EBRs of the giant panda, dog, and cat, respectively. Among these genes, one olfactory receptor gene, *OR6C76*, was identified as located in a giant panda EBR, and four olfactory receptor genes (*OR2G2*, *OR2G3*, *OR4K14*, and *OR4Q2*) were identified as located in cat EBRs. Moreover, we found that the sweet receptor gene, *TAS1R2*, which is located in an EBR of the giant panda genome, exhibits an intact functional homologous gene in dog genome but a homologous pseudogene in the cat genome. The pseudogenization of this sweet-receptor gene accounts for the cat’s indifference toward sugar [34–36]. This suggests that chromosome rearrangement may play a role in the pseudogenization of *TAS1R2* in the cat genome.

It has been demonstrated that the genes located in EBRs are preferentially enriched in specific functional pathways. Larkin et al. [46] performed a synteny analysis among 10 amniote genomes and identified a total of 1064 EBRs. The gene annotations for these EBRs showed that genes associated with the inflammatory response and muscle contractility were enriched. Groenen et al. [45] revealed that porcine EBRs were enriched for genes involved in the sensory perceptions of taste, indicating that taste may be affected by chromosome rearrangement. In this study, we found that some genes located in giant panda, dog, and cat EBRs were functionally enriched in the sensory perception of olfaction. These results suggest that the olfaction phenotype may be affected by chromosome rearrangement events. Previous studies showed that some olfactory proteins have interaction with putative pheromones [47] and some chemical constituents may contribute to successful reproduction of giant panda with a characteristically sophisticated chemical communication system [48]. Our study indicates that the evolution of olfaction system in giant panda may be affected by events associated with chromosome rearrangements.

## Conclusions

Overall, taking advantage of 10X Genomics, flow-sorting, and cross-species chromosome painting, we presented a chromosome-level giant panda genome. Our study provides an effective approach of transforming fragmented scaffold-level assemblies to chromosome-level. Based on chromosome-level giant panda, dog, and cat genomes, we identified some previously undetectable chromosome fission events. The EBR analysis in three Carnivora genomes showed significant increases for gene density, GC content, and repetitive content in giant panda and dog EBRs as compared with their respective whole genomes. The functional enrichment analysis of EBR genes in giant panda, dog, and cat genomes showed that olfaction phenotype may be affected by events associated with chromosome rearrangement, linking chromosome evolution to functional gene evolution.

## Materials and methods

### 10X Genomics library construction and sequencing

The blood sample used for the 10X Genomics library construction was acquired from a male giant panda from the Beijing Zoo. Blood DNA was extracted using a DNeasy Blood and Tissue kit (Qiagen) following the manufacturer’s instructions. The 10X Genomics libraries were constructed with a Chromium™ Genome Library Kit and Gel Bead Kit v2 (10X Genomics) following the manufacturer’s instructions. The libraries were sequenced on the Illumina X-Ten platform to generate 228 Gb paired-end reads with a read length of 150 bp.

### Scaffolding of the draft genome with 10X Genomics linked-reads and dog genome as an assisting reference

A draft genome assembly was first generated using an 82× published paired-end reads (SRX1351594, SRX1352275, to SRX1352277) and mate-pair reads data (SRX007019 to SRX007029). This draft assembly and the 10X Genomics linked-reads were used as the input data for Scaff10X (https://github.com/wtsi-hpag/Scaff10X), a software pipeline specifically designed using 10X Genomics linked-reads to assemble genomes. We did not perform de novo assembly using 10X Genomics linked-reads because the linked-reads were from a male giant panda, different from the original sequenced female individual “Jingjing” [16, 17]. Then, the genome synteny result between dog and giant panda was used to further link giant panda scaffolds that were adjacently aligned to the same dog chromosome. The quality metrics for this new assembly and two previously reported assemblies were obtained using Quast software [49] with the default parameters.

### Whole-genome alignment

The reference genomes of dog and cat were downloaded from Ensembl (http://www.ensembl.org/). The alignment of different scaffolds of the giant panda to the chromosomes of dog or cat genomes was performed using Progressive Mauve software with default parameters [50].

### Flow-sorting and chromosome sequencing

A fibroblast cell line was derived from a male giant panda and cultured in DMEM (Invitrogen, CA) medium supplemented with 15% fetal bovine serum and 500 g/ml of geneticin. The cell line was treated with demecolcine (0.1 g/ml) for 6 h after subculturing for 24 h. Giant panda chromosomes were prepared as previously described [51, 52] and stained overnight with Hoechst 33258 (Sigma, St. Louis, MO) and Chromomycin A3 (Sigma). The stained chromosomes were treated with 25 mM of sodium sulfite an hour before flow-sorting analysis. Stained metaphase chromosome suspensions were analyzed on a flow cytometer (MoFlo, Beckman Coulter) as previously described. The data rate was 10,000~15,000 events/s, with an optimal sheath pressure of ~ 60 psi and a drop drive frequency of ~ 95 kHz, using a 70-μm Cytonozzle tip on the high-purity sort option of the single mode per single drop envelope. The chromosomes were flow-sorted into sterile 500 μl Eppendorf tubes containing 33 μl of sterile UV-treated distilled water. Each of the 22 giant panda chromosomes was individually sorted. Fifty thousand copies of each chromosome were finally collected, and chromosome clumps and debris were carefully excluded. However, chromosome 9 could not be resolved from X, and chromosome 10 was mixed with 11 during flow-sorting.

The chromosomes were amplified using a GenomePlex Complete Whole Genome Amplification (WGA) Kit (WGA2, Sigma-Aldrich) following the protocol provided by the manufacturer. Individual libraries were prepared for the flow-sorted chromosomes with an average insert size of ~ 300 bp and sequenced on the Illumina X-Ten platform with 150 bp paired-end reads. The alignment of the chromosome-derived reads with contigs was used to assign contigs to chromosomes. The final assembly was then assigned to the giant panda chromosomes by mapping the flow-sorted chromosome sequencing reads data.

### Arranging the giant panda scaffolds on chromosomes using cat genome as an assisting reference

In this study, based on the synteny results between giant panda and cat genome, and previous cross-species chromosome paintings [1], we arranged the giant panda scaffolds on the same chromosome. Because the orientation of the giant panda scaffolds was unknown, we sorted the scaffolds based on the directions of the cat chromosomes to maximize collinearity with the cat genome. Finally, we obtained a chromosome-level giant panda genome.

### Repeat masking

Known repeats and low complexity DNA sequences were identified using RepeatMasker version 4.0.7 [53] (http://www.repeatmasker.org/) against the Repbase library (version 20170127). Additionally, repeat elements of the giant panda genome were de novo predicted using RepeatModeler version 1.0.11 [53], and a second round of RepeatMasker was run with the generated model. A PERL script was used to parse the above results (.out file) generated by RepeatMasker to count the number of repetitive sequences.

### Genome annotation

Genome annotation was performed using the genome annotation pipeline Maker [54] version 3.00 with transcriptome alignment, de novo gene prediction, and homology-based gene prediction. Briefly, the transcript sequences of the giant panda downloaded from NCBI were used as EST data. These transcript sequences were assembled using RNA-seq data for 15 samples from 5 giant pandas (2 blood samples from 2 females; 1 blood sample from 1 male; pallium, liver, small intestine, stomach, colon, and testis samples from 1 male adult; skeletal muscle, pituitary, tongue, ovary, and 2 skin tissue samples from 1 female adult) [55]. The longest protein sequences that corresponded to genes from human, dog, and giant panda were used as protein data for Maker. Maker was run with the following parameters: softmask = 1, Augustus_species = human, and min_contig = 10,000.

### Detection of EBR in three Carnivora genomes

To detect potential EBRs, we determined large-scale HSBs based on pairwise whole-genome alignment using the chromosome sequences of three Carnivora genomes. The orthologous protein-coding genes among the three genomes were first obtained using OrthoFinder software [56]. Then, the genome sequence and orthologous protein-coding genes were used as input file for SyMAP software [33] to build large-scale HSBs. Particularly, the SyMAP program first aligned the genomic sequence using MUMmer method [57] to detect raw local synteny blocks which were defined as 20 bp or longer exact matches between two genomes. Then, the raw local synteny blocks were clustered and filtered using the orthologous protein-coding genes to form anchors. The filtered anchors were input into a synteny algorithm to form large-scale HSBs where intervening micro-rearrangements were allowed. Next, we determined the EBRs in three Carnivora genomes based on the large-scale HSBs junctions. The EBR is defined as the interval between two large-scale HSBs that is demarcated by the end-sequence coordinates of large-scale HSBs on each side. The Mann-Whitney *U* test implemented in R (version 3.4.1) was applied to compare the relative gene density, GC content, and repetitive content within the EBRs of each chromosome versus the whole chromosome for each species.

### Annotation of genes located in EBRs

To obtain a better resolution for gene-level analysis, we used the Ensembl biomart gene annotation system (http://asia.ensembl.org/biomart/martview/). For the giant panda, we first aligned the proteins located in EBRs to the whole protein sequences downloaded from Ensembl using the blastp method, and the best alignment was the corresponding Ensembl protein. Then, the Ensembl protein was converted into corresponding Ensembl gene. For the cat and dog, the canonical record for the start position and end position of each EBR was directly used to obtain the Ensembl gene. Subsequently, the set of Ensembl genes were converted to their orthologous human genes. The orthologous human genes were analyzed using the GeneTrail2 (https://genetrail2.bioinf.uni-sb.de/) method to identify Gene Ontology (GO) term [58] and Kyoto Encyclopedia of Genes and Genomes (KEGG) pathway enrichment [59].

#### Review history

The review history is available as Additional file 3.

#### Peer review information

Tim Sands and Barbara Cheifet were the primary handling editors for this manuscript and managed its editorial process and peer review in collaboration with the rest of the editorial team.

## Supplementary information

**Additional file 1: **Supplementary **Figure S1-S4.**, Supplementary **Table S1-S3 and S7-S15.**

**Additional file 2: **Supplementary **Tables S4-S6 and S16-S18.**

**Additional file 3.** Review history.

## Data Availability

The 10X Genomics and flowed-sorted chromosome sequencing data, and the genome assembly generated in this study are available at NCBI under BioProject ID PRJNA588422 [60]. Additionally, these genome sequencing data have also been submitted to BIGD database under BioProject ID PRJCA001903 [61]. The paired-end reads (SRX1351594, SRX1352275, to SRX1352277) and mate-pair reads (SRX007019 to SRX007029) used to generate the primary assembly were downloaded from NCBI Sequence Read Archive (SRA) database.
