# Supplementary material for: Chromosome-level genome assembly for giant panda provides novel insights into Carnivora chromosome evolution
Source: Genome Biol. 2019 Dec 6;20:267. doi: 10.1186/s13059-019-1889-7 (PMC6898958; doi:10.1186/s13059-019-1889-7)
Supplement: Supplementary file 1 — Additional file 1: Supplementary Figure S1-S4., Supplementary Table S1-S3 and S7-S15. [file 13059_2019_1889_MOESM1_ESM.docx]

**Supplementary Information**

# **Chromosome-level genome assembly for giant panda provides novel insights into Carnivora chromosome evolution**

Huizhong Fan^1,2#^, Qi Wu^1#^, Fuwen Wei^1,2,3^, Fengtang Yang^4^, Bee Ling Ng^4^, Yibo Hu^1,3*^

^1^CAS Key Laboratory of Animal Ecology and Conservation Biology, Institute of Zoology, Chinese Academy of Sciences, Beijing, China.

^2^University of Chinese Academy of Sciences, Beijing, China.

^3^Center for Excellence in Animal Evolution and Genetics, Chinese Academy of Sciences, Kunming, China.

^4^Wellcome Trust Sanger Institute, Wellcome Genome Campus, Hinxton, Cambridge, United Kingdom.

^#^These authors contributed equally to this work.

*Correspondence should be addressed to Yibo Hu (ybhu@ioz.ac.cn).

This file includes

Supplementary Figure S1-S4

Supplementary Table S1-S18.

Table S4-S6 and Table S16-S18 are in excel form.

**
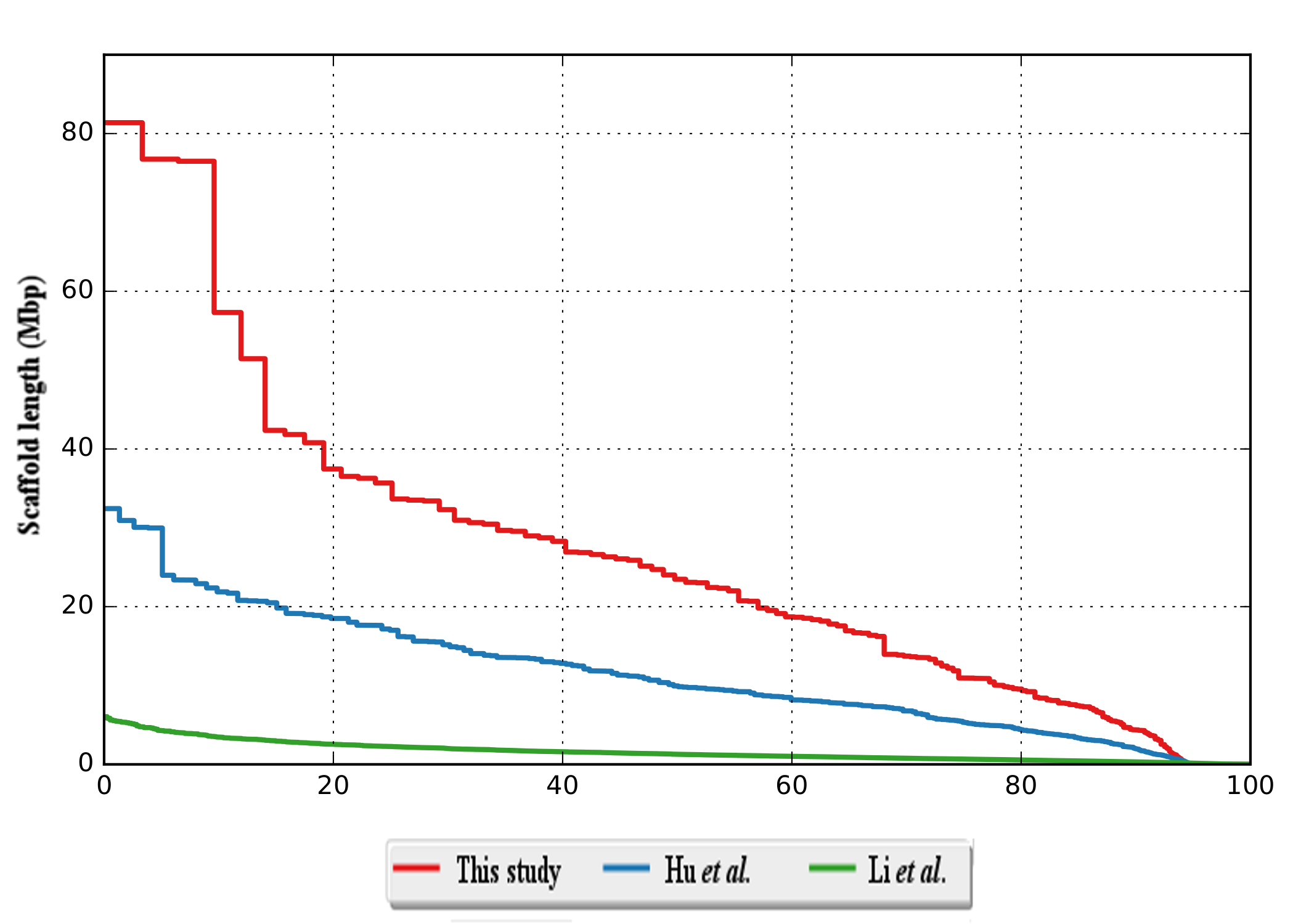
**

**Fig. S1 Distribution of the cumulative scaffold length compared to previously published genome assemblies of the giant panda.** The red line represents the genome assembly obtained in this study. It shows that the greater N50 value of our new assembly is not simply due to a few longer scaffolds than Li et al. [16] and Hu et al. [17].

**
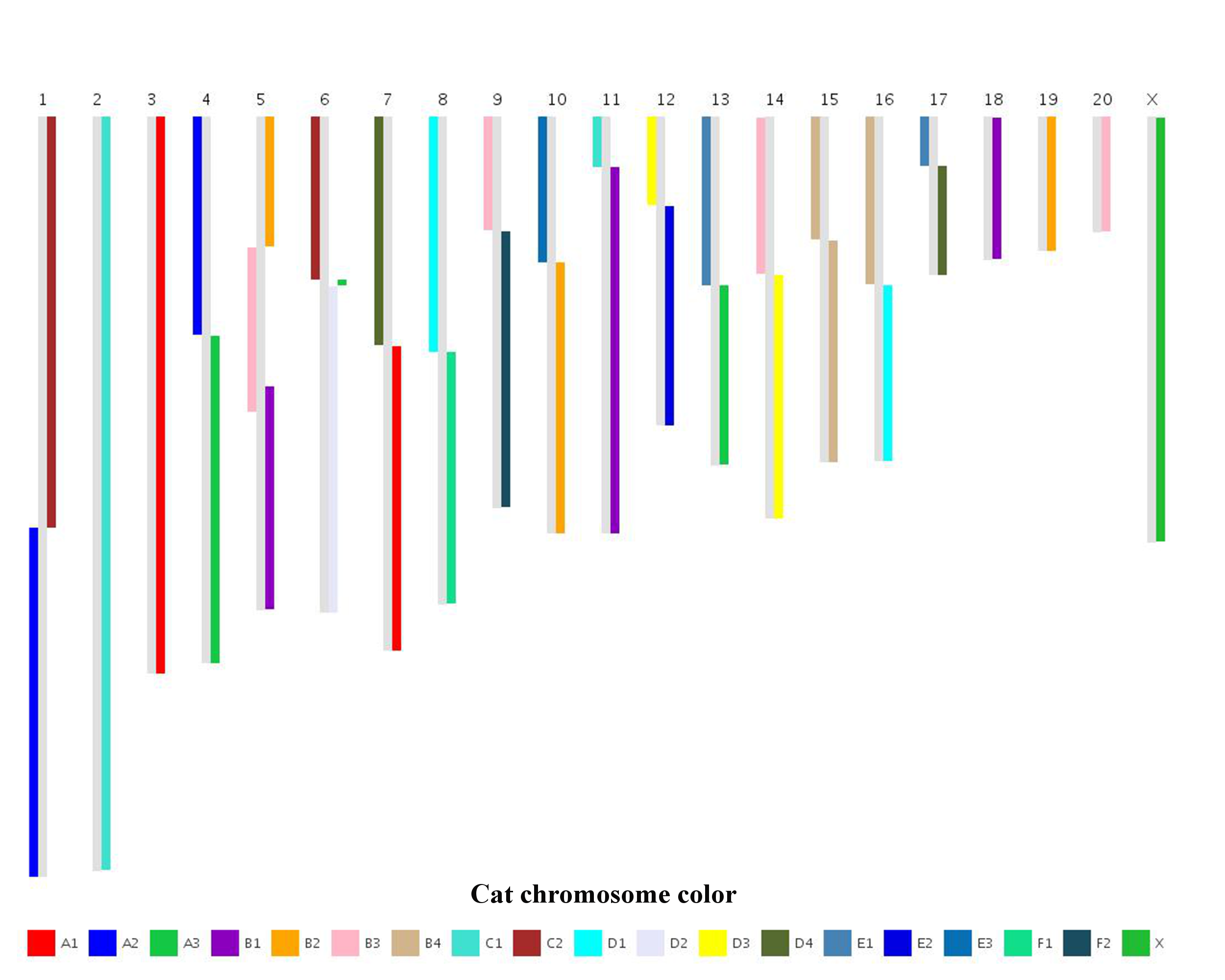
**

**Fig. S2 The large-scale HSBs between cat and giant panda chromosomes detected by SyMAP**. The gray blocks in the middle represent the giant panda chromosomes and the color blocks represent the cat chromosomes.


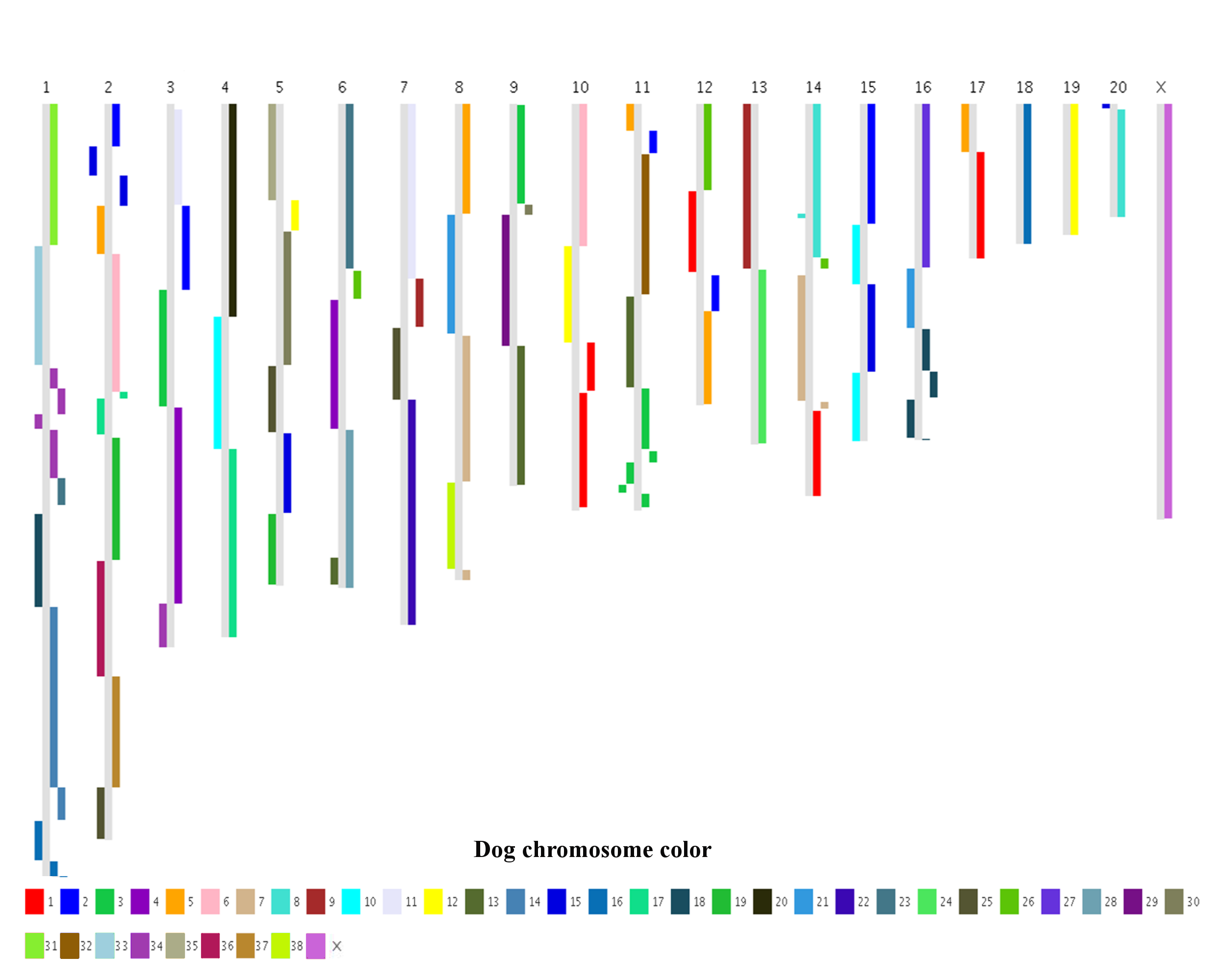


**Fig. S3** **The large-scale HSBs between dog and giant panda chromosomes detected by SyMAP**. The gray blocks in the middle represent the giant panda chromosomes and the color blocks represent the dog chromosomes.


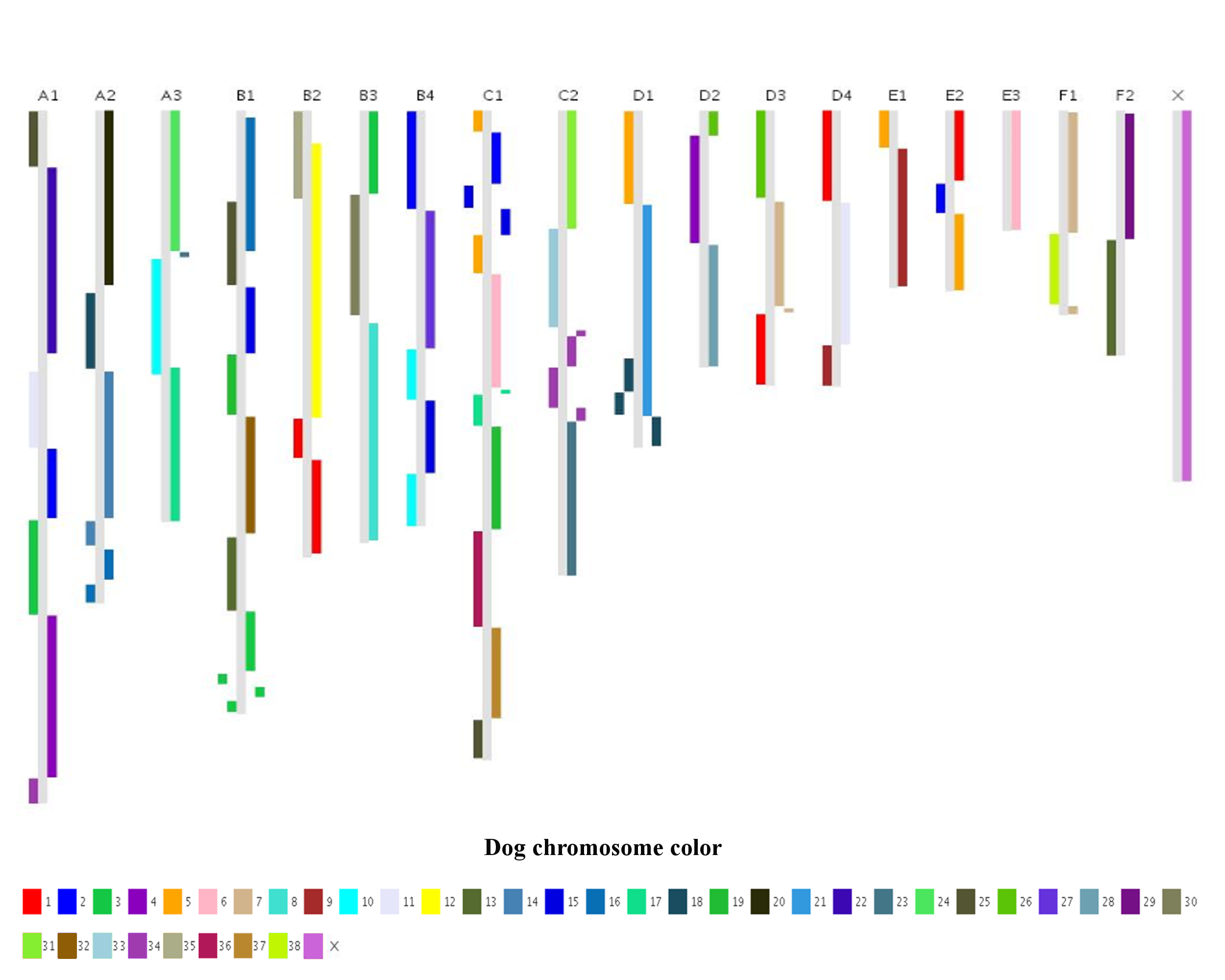


**Fig. S4** **The large-scale HSBs between dog and cat chromosomes detected by SyMAP**. The gray blocks in the middle represent the cat chromosomes and the color blocks represent the dog chromosomes.

**Table S1** Comparison of three steps of giant panda genome assembly metrics.

|  | Mate-pair scaffolding | Add 10X scaffolding | Add dog synteny information |
| --- | --- | --- | --- |
| N50 | 1,239,756 | 18,035,266 | 23,473,669 |
| N75 | 625,637 | 8,423,095 | 10,955,135 |
| L50 | 581 | 40 | 34 |
| L75 | 1,246 | 88 | 70 |
| Number | 81,786 | 78,637 | 77,897 |
| Longest | 7,007,367 | 58,172,990 | 81,377,464 |
| Total Size | 2,444,413,130 | 2,444,700,256 | 2,445,001,150 |
| Total number(≥1Mbp) | 795 | 206 | 152 |
| Total number(≥10Mbp) | 0 | 81 | 77 |

**Table S2** Sequencing and statistics for giant panda chromosomes

| Chromosome | Assembly Size (Mb) | Sequence Size  (Mb) | Coverage (x-fold) | Scaffold Number | Scaffold N50 (Mb) |
| --- | --- | --- | --- | --- | --- |
| Chr1 | 216.058 | 14811.7 | 68.6 | 573 | 26.062 |
| Chr2 | 202.370 | 18796.2 | 92.9 | 579 | 28.739 |
| Chr3 | 153.076 | 10177.5 | 66.5 | 300 | 81.377 |
| Chr4 | 148.548 | 11997.9 | 80.8 | 565 | 24.016 |
| Chr5 | 133.689 | 12189.6 | 91.2 | 451 | 76.490 |
| Chr6 | 133.836 | 13657.7 | 102.0 | 326 | 17.798 |
| Chr7 | 143.433 | 10235.3 | 71.4 | 193 | 37.456 |
| Chr8 | 140.274 | 13822.1 | 98.5 | 706 | 23.023 |
| Chr9-X | 226.786 | 32237.1 | 142.1 | 1469 | 19.122 |
| Chr10-11 | 226.670 | 28025.1 | 123.6 | 1291 | 30.469 |
| Chr12 | 86.496 | 9498.9 | 109.8 | 762 | 9.351 |
| Chr13 | 96.512 | 10531.9 | 109.1 | 813 | 13.950 |
| Chr14 | 109.188 | 16250 | 148.8 | 512 | 16.694 |
| Chr15 | 94.568 | 9245.6 | 97.8 | 789 | 16.221 |
| Chr16 | 93.458 | 9528.8 | 102.0 | 355 | 16.362 |
| Chr17 | 45.560 | 8674.4 | 190.4 | 807 | 28.974 |
| Chr18 | 40.727 | 6616.4 | 162.5 | 218 | 33.405 |
| Chr19 | 39.295 | 8711.3 | 221.7 | 378 | 35.684 |
| Chr20 | 33.757 | 9034.2 | 267.6 | 495 | 29.558 |
| Total | 2364.298 | 254041.7 |  | 11582 |  |

**Table S3 The chromosome fission events identified in this study and in previous published study**

| **Giant panda Chromosome** | **Dog Chromosomes** | | **Cat Chromosomes** | |
| --- | --- | --- | --- | --- |
|  | **This study** | **Nei et al.** | **This study** | **Nei et al.** |
| **Chr1** | 31-33 | 27-30 | C2-A2 | C2p+q-A2q |
| **Chr1** | 33-34 | 30-35 |  |  |
| **Chr1** | 34-23 | 35-18 |  |  |
| **Chr1** | 23-18 |  |  |  |
| **Chr1** | 18-14 | 18-14 |  |  |
| **Chr1** | 14-16 | 14-16 |  |  |
| **Chr2** | 2-15 |  |  |  |
| **Chr2** | 15-5 | 15-5 |  |  |
| **Chr2** | 5-6 | 5-6 |  |  |
| **Chr2** | 6-17 | 6-17 |  |  |
| **Chr2** | 17-19 | 17-19 |  |  |
| **Chr2** | 19-36 | 19-36 |  |  |
| **Chr2** | 36-37 | 36-33 |  |  |
| **Chr2** | 37-25 | 33-28 |  |  |
| **Chr3** | 11-2 | 11-2 |  |  |
| **Chr3** | 2-3 | 2-3 |  |  |
| **Chr3** | 3-4 | 3-4 |  |  |
| **Chr3** | 4-34 | 4-35 |  |  |
| **Chr4** | 20-10 | 20-10 | A2-A3 | A2p-A3q |
| **Chr4** | 10-17 | 10-17 |  |  |
| **Chr5** | 35-12 | 37-25 | B2-B3 | B2p-B3q |
| **Chr5** | 12-30 | 25-28 | B3-B1 | B3q-B1q |
| **Chr5** | 30-25 | 28-15 |  |  |
| **Chr5** | 25-15 | 15-19 |  |  |
| **Chr5** | 15-19 | 19-32 |  |  |
| **Chr6** | 23-26 | 35-23 | C2-A3 | C2q-D2 |
| **Chr6** | 26-4 | 23-4 | A3-D2 |  |
| **Chr6** | 4-28 | 4-31 |  |  |
| **Chr6** | 28-13 |  |  |  |
| **Chr7** | 11-9 | 9-11 | D4-A1 | D4q-A1p |
| **Chr7** | 9-25 | 11-28 |  |  |
| **Chr7** | 25-22 | 28-22 |  |  |
| **Chr8** | 5-21 | 5-21 | D1-F1 | D1p+q-F1 |
| **Chr8** | 21-7 | 21-38 |  |  |
| **Chr8** | 7-38 | 38-7 |  |  |
| **Chr8** | 38-7 |  |  |  |
| **Chr9** | 3-30 | 3-34 | B3-F2 | B3p-F2 |
| **Chr9** | 30-29 | 34-13 |  |  |
| **Chr9** | 29-13 |  |  |  |
| **Chr10** | 6-12 | 6-12 | E3-B2 | E3-B2q |
| **Chr10** | 12-1 | 12-1 |  |  |
| **Chr11** | 5-2 | 5-2 | C1-B1 | C1p-B1q |
| **Chr11** | 2-32 | 2-32 |  |  |
| **Chr11** | 32-13 | 32-13 |  |  |
| **Chr11** | 13-3 | 13-3 |  |  |
| **Chr12** | 26-1 | 26-1 | D3-E2 | D3p-E2 |
| **Chr12** | 1-2 | 1-5 |  |  |
| **Chr12** | 2-5 | 5-2 |  |  |
| **Chr13** | 9-24 | 9-24 | E1-A3 | E1p+q-A3 |
| **Chr14** | 8-26 | 8-25 | B3-D3 | B3q-D3q |
| **Chr14** | 26-7 | 25-7 |  |  |
| **Chr14** | 7-1 | 7-1 |  |  |
| **Chr15** | 2-10 | 2-29 |  |  |
| **Chr15** | 10-15 | 29-10 |  |  |
| **Chr15** | 15-10 | 10-15 |  |  |
| **Chr15** |  | 15-10 |  |  |
| **Chr16** | 27-21 | 2-21 | B4-D1 | B4p-D1q |
| **Chr16** | 21-18 | 21-18 |  |  |
| **Chr17** | 5-1 | 5-1 | E1-D4 | E1p-D4p |
| **Chr20** | 15-8 |  |  |  |

Note: The nomenclature of dog chromosomes is different between the study of Nei et al. [1] and Ensembl dog genome assembly, which leads to the differences in chromosome numbering.

**Table S4** The detailed information of HSBs in giant panda genome.

**Table S5** The detailed information of HSBs in cat genome.

**Table S6** The detailed information of HSBs in dog genome.

**Table S7** The detailed information of identified EBRs caused by inter-chromosome events in giant panda genome.

| **Chromosome** | **Start** | **End** | **Dog chromosome 1** | **Dog chromosome 2** | **Start** | **End** | **Cat**  **chromosome 1** | **Cat**  **chromosome 2** |
| --- | --- | --- | --- | --- | --- | --- | --- | --- |
| **chr1** | 38,422,715 | 38,639,485 | 31 | 33 |  |  |  |  |
| **chr1** | 71,029,452 | 71,889,008 | 33 | 34 |  |  |  |  |
| **chr1** | 101,654,119 | 101,800,562 | 34 | 23 |  |  |  |  |
| **chr1** | 108,935,669 | 111,379,533 | 23 | 18 | 108,890,741 | 109,088,821 | C2 | A2 |
| **chr1** | 136,511,304 | 136,724,449 | 18 | 14 |  |  |  |  |
| **chr1** | 194,410,640 | 194,611,134 | 14 | 16 |  |  |  |  |
| **chr2** | 11,590,774 | 11,711,452 | 2 | 15 |  |  |  |  |
| **chr2** | 27,827,037 | 27,888,818 | 15 | 5 |  |  |  |  |
| **chr2** | 40,750,354 | 40,848,788 | 5 | 6 |  |  |  |  |
| **chr2** | 78,139,213 | 78,342,423 | 6 | 17 |  |  |  |  |
| **chr2** | 89,929,871 | 90,862,054 | 17 | 19 |  |  |  |  |
| **chr2** | 123,756,436 | 124,045,883 | 19 | 36 |  |  |  |  |
| **chr2** | 155,393,172 | 155,524,690 | 36 | 37 |  |  |  |  |
| **chr2** | 185,491,802 | 185,604,593 | 37 | 25 |  |  |  |  |
| **chr3** | 27,499,484 | 27,734,982 | 11 | 2 |  |  |  |  |
| **chr3** | 50,681,130 | 50,746,324 | 2 | 3 |  |  |  |  |
| **chr3** | 82,347,705 | 82,487,482 | 3 | 4 |  |  |  |  |
| **chr3** | 135,681,299 | 135,741,543 | 4 | 34 |  |  |  |  |
| **chr4** | 57,891,952 | 57,951,233 | 20 | 10 | 57,934,601 | 58,205,424 | A2 | A3 |
| **chr4** | 93,649,490 | 93,770,202 | 10 | 17 |  |  |  |  |
| **chr5** | 26,172,289 | 26,214,440 | 35 | 12 |  |  |  |  |
| **chr5** | 34,598,264 | 34,758,533 | 12 | 30 | 34,630,828 | 34,804,519 | B2 | B3 |
| **chr5** | 70,993,666 | 71,235,052 | 30 | 25 |  |  |  |  |
| **chr5** | 89,289,924 | 89,504,986 | 25 | 15 |  |  |  |  |
| **chr5** | 111,134,690 | 111,516,538 | 15 | 19 |  |  |  |  |
| **chr6** |  |  |  |  | 43,185,477 | 43,362,607 | C2 | A3 |
| **chr6** | 44,815,023 | 45,363,271 | 23 | 26 | 44,749,889 | 45,003,918 | A3 | D2 |
| **chr6** | 52,950,608 | 53,205,586 | 26 | 4 |  |  |  |  |
| **chr6** | 88,445,714 | 88,691,482 | 4 | 28 |  |  |  |  |
| **chr7** | 47,658,815 | 47,667,277 | 11 | 9 |  |  |  |  |
| **chr7** | 60,692,285 | 60,885,264 | 9 | 25 | 60,687,970 | 61,019,109 | D4 | A1 |
| **chr7** | 80,279,196 | 80,386,634 | 25 | 22 |  |  |  |  |
| **chr8** | 29,834,285 | 30,150,998 | 5 | 21 |  |  |  |  |
| **chr8** | 62,485,768 | 62,967,319 | 21 | 7 | 62,426,842 | 62,571,942 | D1 | F1 |
| **chr8** | 102,635,762 | 102,837,243 | 7 | 38 |  |  |  |  |
| **chr8** | 126,305,898 | 126,495,674 | 38 | 7 |  |  |  |  |
| **chr9** | 27,110,714 | 27,404,900 | 3 | 30 |  |  |  |  |
| **chr9** | 30,172,322 | 30,344,168 | 30 | 29 | 30,097,251 | 30,495,477 | B3 | F2 |
| **chr9** | 65,698,789 | 65,837,984 | 29 | 13 |  |  |  |  |
| **chr10** | 38,667,082 | 38,898,144 | 6 | 12 | 38,711,269 | 38,899,630 | E3 | B2 |
| **chr10** | 64,902,428 | 65,035,500 | 12 | 1 |  |  |  |  |
| **chr11** | 7,388,481 | 7,556,234 | 5 | 2 |  |  |  |  |
| **chr11** | 13,544,756 | 13,711,345 | 2 | 32 | 13,545,715 | 13,658,900 | C1 | B1 |
| **chr11** | 51,723,228 | 52,413,162 | 32 | 13 |  |  |  |  |
| **chr11** | 77,077,692 | 77,221,214 | 13 | 3 |  |  |  |  |
| **chr12** | 23,678,413 | 23,958,099 | 26 | 1 | 23,625,439 | 23,862,102 | D3 | E2 |
| **chr12** | 45,892,914 | 46,645,055 | 1 | 2 |  |  |  |  |
| **chr12** | 56,241,423 | 56,326,249 | 2 | 5 |  |  |  |  |
| **chr13** | 44,893,842 | 44,991,656 | 9 | 24 | 44,863,399 | 44,918,750 | E1 | A3 |
| **chr14** | 41,765,425 | 41,952,491 | 8 | 26 | 41,762,568 | 41,951,798 | B3 | D3 |
| **chr14** | 44,747,808 | 46,577,765 | 26 | 7 |  |  |  |  |
| **chr14** | 82,870,071 | 83,321,186 | 7 | 1 |  |  |  |  |
| **chr15** | 32,709,814 | 33,112,461 | 2 | 10 |  |  |  |  |
| **chr15** | 49,111,124 | 49,172,295 | 10 | 15 |  |  |  |  |
| **chr15** | 72,845,205 | 73,123,026 | 15 | 10 |  |  |  |  |
| **chr16** | 44,606,990 | 44,845,231 | 27 | 21 | 44,630,748 | 44,814,958 | B4 | D1 |
| **chr16** | 61,093,600 | 61,247,072 | 21 | 18 |  |  |  |  |
| **chr17** | 13,179,199 | 13,304,038 | 5 | 1 | 13,173,805 | 13,349,618 | E1 | D4 |
| **chr20** | 1,269,372 | 1,649,254 | 15 | 8 |  |  |  |  |

**Table S8** The detailed information of identified EBRs caused by inter-chromosome events in cat genome.

| **Chromosome** | **Start** | **End** | **Dog Chromosome 1** | **Dog chromosome 2** | **Start** | **End** | **Panda chromosome 1** | **Panda chromosome 2** |
| --- | --- | --- | --- | --- | --- | --- | --- | --- |
| A1 | 19,268,001 | 19,863,669 | 25 | 22 |  |  |  |  |
| A1 | 83,246,790 | 89,327,233 | 22 | 11 | 83,237,296 | 84,003,326 | 7 | 3 |
| A1 | 115,553,227 | 115,977,449 | 11 | 2 |  |  |  |  |
| A1 | 139,566,782 | 140,155,555 | 2 | 3 |  |  |  |  |
| A1 | 172,553,727 | 172,984,324 | 3 | 4 |  |  |  |  |
| A1 | 228,155,429 | 228,635,209 | 4 | 34 |  |  |  |  |
| A2 | 60,087,973 | 62,581,700 | 20 | 18 | 60,088,374 | 60,793,987 | 4 | 1 |
| A2 | 88,315,133 | 89,506,556 | 18 | 14 |  |  |  |  |
| A2 | 148,851,264 | 150,327,464 | 14 | 16 |  |  |  |  |
| A3 | 48,319,548 | 48,580,552 | 24 | 23 | 48,368,143 | 48,609,976 | 13 | 6 |
| A3 | 50,344,719 | 50,965,452 | 23 | 10 | 50,333,704 | 50,994,002 | 6 | 4 |
| B1 | 60,045,167 | 60,658,410 | 25 | 15 |  |  |  |  |
| B1 | 83,375,786 | 83,547,086 | 15 | 19 |  |  |  |  |
| B1 | 104,279,519 | 104,990,092 | 19 | 32 | 104,267,448 | 104,768,899 | 5 | 11 |
| B1 | 144,764,809 | 145,999,771 | 32 | 13 |  |  |  |  |
| B1 | 171,293,638 | 171,532,030 | 13 | 3 |  |  |  |  |
| B2 |  |  |  |  | 38,702,341 | 39,659,829 | 5 | 19 |
| B2 |  |  |  |  | 77,531,942 | 77,992,746 | 19 | 10 |
| B2 | 105,185,820 | 105,434,246 | 12 | 1 |  |  |  |  |
| B3 | 28,557,952 | 28,859,069 | 3 | 30 |  |  |  |  |
| B3 |  |  |  |  | 32,010,123 | 32,417,366 | 9 | 5 |
| B3 | 70,352,889 | 72,920,124 | 30 | 8 | 70,211,684 | 70,868,784 | 5 | 20 |
| B3 |  |  |  |  | 103,529,936 | 104,271,411 | 20 | 14 |
| B4 | 33,901,165 | 34,387,482 | 2 | 27 | 33,916,160 | 34,318,007 | 15 | 16 |
| B4 | 81,460,702 | 81,994,412 | 27 | 10 | 81,994,321 | 81,994,360 | 16 | 15 |
| B4 | 98,905,097 | 99,367,124 | 10 | 15 |  |  |  |  |
| B4 | 124,304,243 | 124,492,232 | 15 | 10 |  |  |  |  |
| C1 | 7,239,729 | 7,740,527 | 5 | 2 |  |  |  |  |
| C1 |  |  |  |  | 13,525,204 | 13,753,957 | 11 | 2 |
| C1 | 25,345,638 | 26,073,892 | 2 | 15 |  |  |  |  |
| C1 | 42,730,753 | 42,868,092 | 15 | 5 |  |  |  |  |
| C1 | 55,936,081 | 56,101,031 | 5 | 6 |  |  |  |  |
| C1 | 94,990,456 | 95,600,820 | 6 | 17 |  |  |  |  |
| C1 | 107,963,929 | 108,263,269 | 17 | 19 |  |  |  |  |
| C1 | 143,481,943 | 143,990,466 | 19 | 36 |  |  |  |  |
| C1 | 176,684,046 | 176,908,290 | 36 | 37 |  |  |  |  |
| C1 | 207,901,614 | 208,500,203 | 37 | 25 |  |  |  |  |
| C2 | 40,786,697 | 40,840,641 | 31 | 33 |  |  |  |  |
| C2 | 74,465,057 | 75,234,503 | 33 | 34 |  |  |  |  |
| C2 | 106,278,548 | 106,558,802 | 34 | 23 |  |  |  |  |
| C2 |  |  |  |  | 114,170,132 | 114,405,952 | 1 | 6 |
| D1 | 32,008,657 | 32,621,642 | 5 | 21 |  |  |  |  |
| D1 |  |  |  |  | 67,266,185 | 67,754,071 | 8 | 16 |
| D2 | 8,659,723 | 8,920,611 | 26 | 4 |  |  |  |  |
| D2 | 45,623,515 | 46,189,255 | 4 | 28 |  |  |  |  |
| D3 | 29,891,368 | 31,350,284 | 26 | 7 |  |  |  |  |
| D3 | 69,314,380 | 70,048,083 | 7 | 1 |  |  |  |  |
| D4 | 31,230,564 | 31,761,071 | 1 | 11 | 31,230,445 | 31,651,132 | 17 | 7 |
| D4 | 80,235,542 | 80,644,881 | 11 | 9 |  |  |  |  |
| E1 | 12,710,947 | 13,106,816 | 5 | 9 | 12,704,666 | 13,114,814 | 17 | 13 |
| E2 | 24,347,715 | 25,103,961 | 1 | 2 |  |  |  |  |
| E2 | 35,328,761 | 35,452,711 | 2 | 5 |  |  |  |  |
| F1 | 42,082,158 | 42,498,307 | 7 | 38 |  |  |  |  |
| F1 | 66,596,921 | 67,251,116 | 38 | 7 |  |  |  |  |
| F2 | 44,225,885 | 44,448,625 | 29 | 13 |  |  |  |  |

**Table S9** The detailed information of identified EBRs caused by inter-chromosome events in dog genome.

| **Chromosome** | **Start** | **End** | **Giant panda chromosome 1** | **Giant panda chromosome 2** | **Start** | **End** | **Cat**  **chromosome 1** | **Cat**  **chromosome 2** |
| --- | --- | --- | --- | --- | --- | --- | --- | --- |
| chr1 | 23,992,757 | 25,046,954 | 14 | 10 | 23,781,964 | 25,101,290 | D3 | B2 |
| chr1 | 69,514,092 | 69,826,501 | 10 | 17 | 69,410,590 | 69,824,720 | B2 | D4 |
| chr1 | 99,327,212 | 99,525,737 | 17 | 12 | 99,230,839 | 99,486,211 | D4 | E2 |
| chr2 | 34,499,421 | 34,868,470 | 15 | 3 | 34,510,958 | 34,895,092 | B4 | A1 |
| chr2 | 57,598,980 | 58,071,002 | 3 | 12 | 57,216,780 | 57,974,351 | A1 | E2 |
| chr2 | 67,640,703 | 67,986,978 | 12 | 2 | 67,844,694 | 68,049,480 | E2 | C1 |
| chr2 | 79,671,658 | 79,809,455 | 2 | 11 |  |  |  |  |
| chr3 | 31,225,943 | 31,748,819 | 3 | 9 | 30,994,418 | 31,591,175 | A1 | B3 |
| chr3 | 58,264,422 | 58,554,168 | 9 | 11 | 58,434,744 | 58,699,426 | B3 | B1 |
| chr4 | 35,078,995 | 35,319,414 | 6 | 3 | 34,991,294 | 35,374,272 | D2 | A1 |
| chr5 | 29,876,442 | 30,182,826 | 8 | 17 | 29,854,847 | 30,146,576 | D1 | E1 |
| chr5 | 42,808,463 | 43,185,149 | 17 | 2 | 42,807,575 | 43,195,065 | E1 | C1 |
| chr5 | 56,051,574 | 56,184,819 | 2 | 11 |  |  |  |  |
| chr5 | 63,420,507 | 63,629,346 | 11 | 12 | 63,350,234 | 63,633,623 | C1 | E2 |
| chr6 | 40,371,257 | 40,737,000 | 10 | 2 | 40,412,555 | 40,673,229 | E3 | C1 |
| chr7 | 43,386,577 | 43,871,874 | 8 | 14 | 43,372,440 | 43,836,579 | F1 | D3 |
| chr9 | 47,742,459 | 47,859,077 | 13 | 7 | 47,724,164 | 47,888,291 | E1 | D4 |
| chr10 | 33,833,627 | 34,248,376 | 15 | 4 | 33,957,467 | 34,220,558 | B4 | A3 |
| chr11 | 26,750,862 | 26,986,372 | 3 | 7 | 26,583,752 | 27,029,164 | A1 | D4 |
| chr12 | 8,820,518 | 9,788,161 | 5 | 19 |  |  |  |  |
| chr12 | 45,699,329 | 46,054,591 | 19 | 10 |  |  |  |  |
| chr13 | 37,032,618 | 38,554,965 | 6 | 11 | 38,146,656 | 38,844,671 | F2 | B1 |
| chr15 | 16,983,710 | 17,320,344 | 2 | 20 | 16,895,216 | 18,752,218 | C1 | B4 |
| chr15 | 18,461,755 | 18,749,453 | 20 | 15 |  |  |  |  |
| chr15 | 42,575,397 | 42,810,940 | 15 | 5 | 42,476,493 | 42,882,599 | B4 | B1 |
| chr16 | 20,946,934 | 21,610,020 | 1 | 18 | 21,090,033 | 21,700,018 | A2 | B1 |
| chr17 | 51,464,469 | 51,566,027 | 4 | 2 | 51,469,986 | 51,571,025 | A3 | C1 |
| chr18 | 24,842,972 | 25,377,935 | 1 | 16 | 24,418,021 | 25,965,544 | A2 | D1 |
| chr19 | 19,530,119 | 21,269,835 | 5 | 2 | 19,532,544 | 20,512,245 | B1 | C1 |
| chr21 | 33,861,813 | 34,348,042 | 8 | 16 |  |  |  |  |
| chr23 | 45,006,928 | 45,093,231 | 6 | 1 |  |  |  |  |
| chr25 | 19,004,379 | 19,193,137 | 7 | 5 | 18,813,034 | 19,196,010 | A1 | B1 |
| chr25 | 37,109,703 | 37,645,180 | 5 | 2 | 36,766,360 | 37,973,675 | B1 | C1 |
| chr26 |  |  |  |  | 31,064,980 | 31,553,783 | D3 | D2 |
| chr26 | 24,956,596 | 27,435,069 | 12 | 14 |  |  |  |  |
| chr30 | 37,149,741 | 37,348,638 | 5 | 9 |  |  |  |  |
| chr34 | 12,100,641 | 12,631,433 | 3 | 1 | 12,016,953 | 12,654,322 | A1 | C2 |

**Table S10** The detailed position of identified EBRs in giant panda, dog and cat genomes.

| **Giant panda** | | | **Dog** | | | **Cat** | | |  | |  |
| --- | --- | --- | --- | --- | --- | --- | --- | --- | --- | --- | --- |
| **Chromosome** | **start** | **end** | **Chromosome** | **start** | **end** | **Chromosome** | **start** | **end** | |  |  |
| **chr1** | 38,422,715 | 38,639,485 | chr1 | 23,781,964 | 25,101,290 | A1 | 19,268,001 | 19,863,669 | |  |  |
| **chr1** | 71,029,452 | 71,889,008 | chr1 | 69,410,590 | 69,826,501 | A1 | 83,237,296 | 89,327,233 | |  |  |
| **chr1** | 101,654,119 | 101,800,562 | chr1 | 99,230,839 | 99,525,737 | A1 | 115,553,227 | 115,977,449 | |  |  |
| **chr1** | 108,890,741 | 111,379,533 | chr2 | 34,499,421 | 34,895,092 | A1 | 139,566,782 | 140,155,555 | |  |  |
| **chr1** | 136,511,304 | 136,724,449 | chr2 | 57,216,780 | 58,071,002 | A1 | 172,553,727 | 172,984,324 | |  |  |
| **chr1** | 194,410,640 | 194,611,134 | chr2 | 67,640,703 | 68,049,480 | A1 | 228,155,429 | 228,635,209 | |  |  |
| **chr2** | 11,590,774 | 11,711,452 | chr2 | 79,671,658 | 79,809,455 | A2 | 60,087,973 | 62,581,700 | |  |  |
| **chr2** | 27,827,037 | 27,888,818 | chr3 | 30,994,418 | 31,748,819 | A2 | 88,315,133 | 89,506,556 | |  |  |
| **chr2** | 40,750,354 | 40,848,788 | chr3 | 58,264,422 | 58,699,426 | A2 | 148,851,264 | 150,327,464 | |  |  |
| **chr2** | 78,139,213 | 78,342,423 | chr4 | 34,991,294 | 35,374,272 | A3 | 48,319,548 | 48,609,976 | |  |  |
| **chr2** | 89,929,871 | 90,862,054 | chr5 | 29,854,847 | 30,182,826 | A3 | 50,333,704 | 50,994,002 | |  |  |
| **chr2** | 123,756,436 | 124,045,883 | chr5 | 42,807,575 | 43,195,065 | B1 | 60,045,167 | 60,658,410 | |  |  |
| **chr2** | 155,393,172 | 155,524,690 | chr5 | 56,051,574 | 56,184,819 | B1 | 83,375,786 | 83,547,086 | |  |  |
| **chr2** | 185,491,802 | 185,604,593 | chr5 | 63,350,234 | 63,633,623 | B1 | 104,267,448 | 104,990,092 | |  |  |
| **chr3** | 27,499,484 | 27,734,982 | chr6 | 40,371,257 | 40,737,000 | B1 | 144,764,809 | 145,999,771 | |  |  |
| **chr3** | 50,681,130 | 50,746,324 | chr7 | 43,372,440 | 43,871,874 | B1 | 171,293,638 | 171,532,030 | |  |  |
| **chr3** | 82,347,705 | 82,487,482 | chr9 | 47,724,164 | 47,888,291 | B2 | 38,702,341 | 39,659,829 | |  |  |
| **chr3** | 135,681,299 | 135,741,543 | chr10 | 33,833,627 | 34,248,376 | B2 | 77,531,942 | 77,992,746 | |  |  |
| **chr4** | 57,891,952 | 58,205,424 | chr11 | 26,583,752 | 27,029,164 | B2 | 105,185,820 | 105,434,246 | |  |  |
| **chr4** | 93,649,490 | 93,770,202 | chr12 | 8,820,518 | 9,788,161 | B3 | 28,557,952 | 28,859,069 | |  |  |
| **chr5** | 26,172,289 | 26,214,440 | chr12 | 45,699,329 | 46,054,591 | B3 | 32,010,123 | 32,417,366 | |  |  |
| **chr5** | 34,598,264 | 34,804,519 | chr13 | 37,032,618 | 38,844,671 | B3 | 70,211,684 | 72,920,124 | |  |  |
| **chr5** | 70,993,666 | 71,235,052 | chr15 | 16,895,216 | 18,752,218 | B3 | 103,529,936 | 104,271,411 | |  |  |
| **chr5** | 89,289,924 | 89,504,986 | chr15 | 18,461,755 | 18,749,453 | B4 | 33,901,165 | 34,387,482 | |  |  |
| **chr5** | 111,134,690 | 111,516,538 | chr15 | 42,476,493 | 42,882,599 | B4 | 81,460,702 | 81,994,412 | |  |  |
| **chr6** | 43,185,477 | 43,362,607 | chr16 | 20,946,934 | 21,700,018 | B4 | 98,905,097 | 99,367,124 | |  |  |
| **chr6** | 44,749,889 | 45,363,271 | chr17 | 51,464,469 | 51,571,025 | B4 | 124,304,243 | 124,492,232 | |  |  |
| **chr6** | 52,950,608 | 53,205,586 | chr18 | 24,418,021 | 25,965,544 | C1 | 7,239,729 | 7,740,527 | |  |  |
| **chr6** | 88,445,714 | 88,691,482 | chr19 | 19,530,119 | 21,269,835 | C1 | 13,525,204 | 13,753,957 | |  |  |
| **chr7** | 47,658,815 | 47,667,277 | chr21 | 33,861,813 | 34,348,042 | C1 | 25,345,638 | 26,073,892 | |  |  |
| **chr7** | 60,687,970 | 61,019,109 | chr23 | 45,006,928 | 45,093,231 | C1 | 42,730,753 | 42,868,092 | |  |  |
| **chr7** | 80,279,196 | 80,386,634 | chr25 | 18,813,034 | 19,196,010 | C1 | 55,936,081 | 56,101,031 | |  |  |
| **chr8** | 29,834,285 | 30,150,998 | chr25 | 36,766,360 | 37,973,675 | C1 | 94,990,456 | 95,600,820 | |  |  |
| **chr8** | 62,426,842 | 62,967,319 | chr26 | 31,064,980 | 31,553,783 | C1 | 107,963,929 | 108,263,269 | |  |  |
| **chr8** | 102,635,762 | 102,837,243 | chr26 | 24,956,596 | 27,435,069 | C1 | 143,481,943 | 143,990,466 | |  |  |
| **chr8** | 126,305,898 | 126,495,674 | chr30 | 37,149,741 | 37,348,638 | C1 | 176,684,046 | 176,908,290 | |  |  |
| **chr9** | 27,110,714 | 27,404,900 | chr34 | 12,016,953 | 12,654,322 | C1 | 207,901,614 | 208,500,203 | |  |  |
| **chr9** | 30,097,251 | 30,495,477 |  |  |  | C2 | 40,786,697 | 40,840,641 | |  |  |
| **chr9** | 65,698,789 | 65,837,984 |  |  |  | C2 | 74,465,057 | 75,234,503 | |  |  |
| **chr10** | 38,667,082 | 38,899,630 |  |  |  | C2 | 106,278,548 | 106,558,802 | |  |  |
| **chr10** | 64,902,428 | 65,035,500 |  |  |  | C2 | 114,170,132 | 114,405,952 | |  |  |
| **chr11** | 7,388,481 | 7,556,234 |  |  |  | D1 | 32,008,657 | 32,621,642 | |  |  |
| **chr11** | 13,544,756 | 13,711,345 |  |  |  | D1 | 67,266,185 | 67,754,071 | |  |  |
| **chr11** | 51,723,228 | 52,413,162 |  |  |  | D2 | 8,659,723 | 8,920,611 | |  |  |
| **chr11** | 77,077,692 | 77,221,214 |  |  |  | D2 | 45,623,515 | 46,189,255 | |  |  |
| **chr12** | 23,625,439 | 23,958,099 |  |  |  | D3 | 29,891,368 | 31,350,284 | |  |  |
| **chr12** | 45,892,914 | 46,645,055 |  |  |  | D3 | 69,314,380 | 70,048,083 | |  |  |
| **chr12** | 56,241,423 | 56,326,249 |  |  |  | D4 | 31,230,445 | 31,761,071 | |  |  |
| **chr13** | 44,863,399 | 44,991,656 |  |  |  | D4 | 80,235,542 | 80,644,881 | |  |  |
| **chr14** | 41,762,568 | 41,952,491 |  |  |  | E1 | 12,704,666 | 13,114,814 | |  |  |
| **chr14** | 44,747,808 | 46,577,765 |  |  |  | E2 | 24,347,715 | 25,103,961 | |  |  |
| **chr14** | 82,870,071 | 83,321,186 |  |  |  | E2 | 35,328,761 | 35,452,711 | |  |  |
| **chr15** | 32,709,814 | 33,112,461 |  |  |  | F1 | 42,082,158 | 42,498,307 | |  |  |
| **chr15** | 49,111,124 | 49,172,295 |  |  |  | F1 | 66,596,921 | 67,251,116 | |  |  |
| **chr15** | 72,845,205 | 73,123,026 |  |  |  | F2 | 44,225,885 | 44,448,625 | |  |  |
| **chr16** | 44,606,990 | 44,845,231 |  |  |  |  |  |  | |  |  |
| **chr16** | 61,093,600 | 61,247,072 |  |  |  |  |  |  | |  |  |
| **chr17** | 13,173,805 | 13,349,618 |  |  |  |  |  |  | |  |  |
| **chr20** | 1,269,372 | 1,649,254 |  |  |  |  |  |  | |  |  |

**Table S11** The information of EBRs in three Carnivora genomes

| **Species** | **EBR Number** | **Total Length (Mb)** | **Maximum Length (Mb)** | **Minimum Length (kp)** | **Mean Length (Kb)** | **Median Length (Kb)** |
| --- | --- | --- | --- | --- | --- | --- |
| **Giant panda** | 59 | 18.91 | 2.49 | 8.46 | 320.45 | 206.25 |
| **Dog** | 37 | 24.21 | 2.48 | 86.30 | 654.58 | 414.75 |
| **Cat** | 55 | 38.18 | 6.09 | 53.95 | 694.20 | 487.89 |

**Table S12** Detailed composition of repeat elements in the EBRs of three carnivore genomes

| **Repeat elements** | **Giant panda** | | | **Cat** | | | **Dog** | | |
| --- | --- | --- | --- | --- | --- | --- | --- | --- | --- |
|  | **Number** | **Length （bp）** | **Percent （%）** | **Number** | **Length （bp）** | **Percent （%）** | **Number** | **Length （bp）** | **Percent （%）** |
| **SINE** | 9,414 | 1,500,172 | 7.93 | 22,042 | 3,407,251 | 8.92 | 14,417 | 2,312,381 | 9.55 |
| SINE/MIR | 3,287 | 473,513 | 2.5 | 5,546 | 824,861 | 2.16 | 2,804 | 404,541 | 1.67 |
| **LINE** | 7,265 | 4,383,369 | 23.18 | 12,291 | 8,520,037 | 22.31 | 8,753 | 6,419,734 | 26.51 |
| LINE/LINE1 | 4,802 | 3,774,555 | 19.96 | 7,884 | 7,357,542 | 19.27 | 6,587 | 5,886,452 | 24.3 |
| LINE/LINE2 | 2,100 | 538,673 | 2.85 | 3,718 | 1,018,679 | 2.67 | 1,879 | 476,798 | 1.97 |
| **LTR elements** | 2,544 | 1,025,346 | 5.42 | 3,891 | 1,700,224 | 4.45 | 2,698 | 1,123,826 | 4.64 |
| LTR/ERVL | 767 | 361,283 | 1.91 | 1,105 | 509,551 | 1.33 | 783 | 321,273 | 1.33 |
| LTR/ERVL-MaLRs | 1,029 | 352,512 | 1.86 | 1,634 | 584,726 | 1.53 | 1,069 | 354,553 | 1.46 |
| **DNA elements** | 2,321 | 493,685 | 2.61 | 3,884 | 803,953 | 2.11 | 2,043 | 417,237 | 1.72 |
| **Simple repeats** | 4,278 | 211,394 | 1.12 | 11,867 | 632,833 | 1.66 | 7,760 | 363,099 | 1.5 |
| **Total** |  | 7,681,590 | 40.63 |  |  |  | 39.88 | 10,928,591 | 45.12 |

**Table S13** Analysis of gene density, GC content and repetitive content for each chromosome in EBRs and whole genome in giant panda.

| **Chromosome** | **Mean gene density** | | **Percentage of GC content (%)** | | **Percentage of Repetitive content (%)** | |
| --- | --- | --- | --- | --- | --- | --- |
|  | **EBRs** | **Genome** | **EBRs** | **Genome** | **EBRs** | **Genome** |
| **Chr1** | 15.76 | 7.07 | 44.04 | 39.80 | 45.36 | 38.67 |
| **Chr2** | 10.02 | 9.00 | 48.73 | 40.93 | 37.14 | 40.42 |
| **Chr3** | 31.97 | 7.36 | 48.30 | 40.39 | 33.25 | 39.18 |
| **Chr4** | 16.64 | 12.18 | 41.20 | 43.28 | 52.88 | 38.97 |
| **Chr5** | 18.45 | 9.09 | 43.08 | 40.49 | 37.90 | 41.66 |
| **Chr6** | 8.76 | 8.18 | 42.11 | 42.35 | 49.80 | 40.16 |
| **Chr7** | 29.27 | 7.43 | 40.21 | 40.61 | 45.78 | 38.50 |
| **Chr8** | 14.95 | 11.52 | 42.85 | 42.23 | 45.68 | 40.43 |
| **Chr9** | 17.18 | 6.62 | 44.61 | 41.33 | 42.36 | 40.53 |
| **Chr10** | 27.51 | 10.70 | 55.41 | 42.34 | 15.34 | 38.25 |
| **Chr11** | 11.27 | 7.62 | 41.99 | 40.68 | 44.41 | 39.85 |
| **Chr12** | 26.09 | 19.07 | 42.63 | 46.58 | 44.23 | 38.70 |
| **Chr13** | 31.24 | 16.18 | 38.36 | 45.90 | 45.08 | 37.70 |
| **Chr14** | 17.82 | 8.14 | 50.37 | 42.17 | 41.55 | 37.75 |
| **Chr15** | 28.83 | 8.48 | 45.39 | 41.89 | 44.78 | 39.67 |
| **Chr16** | 23.54 | 15.80 | 40.33 | 42.53 | 51.75 | 39.66 |
| **Chr17** | 12.02 | 11.12 | 41.85 | 44.32 | 39.81 | 39.72 |
| **Chr18** |  | 6.30 |  | 39.62 |  | 40.29 |
| **Chr19** |  | 7.04 |  | 39.00 |  | 40.76 |
| **Chr20** | 83.17 | 10.92 | 44.20 | 38.99 | 40.07 | 41.43 |
| **ChrX** |  | 7.84 |  | 40.61 |  | 53.17 |
| **Mean** | 23.58 | 9.89 | 44.20 | 41.72 | 42.07 | 40.26 |

**Table S14** Analysis of gene density, GC content and repetitive content for each chromosome in EBRs and whole genome in dog genome.

| **Chromosome** | **Mean gene density** | | **Percentage of GC content (%)** | | **Percentage of Repetitive content (%)** | |
| --- | --- | --- | --- | --- | --- | --- |
|  | **EBRs** | **Genome** | **EBRs** | **Genome** | **EBRs** | **Genome** |
| **Chr1** | 14.78 | 9.94 | 42.85 | 41.38 | 50.76 | 42.08 |
| **Chr2** | 15.59 | 8.65 | 41.64 | 42.54 | 52.67 | 43.15 |
| **Chr3** | 16.82 | 5.43 | 44.44 | 40.15 | 51.25 | 43.03 |
| **Chr4** | 15.67 | 5.97 | 47.66 | 40.24 | 50.03 | 42.52 |
| **Chr5** | 13.25 | 11.07 | 43.78 | 44.01 | 49.61 | 40.50 |
| **Chr6** | 43.75 | 11.32 | 46.05 | 42.36 | 52.19 | 43.17 |
| **Chr7** | 36.04 | 8.04 | 47.17 | 40.82 | 38.39 | 42.50 |
| **Chr8** | - | 8.22 | - | 40.44 | - | 43.40 |
| **Chr9** | 12.19 | 18.11 | 47.26 | 45.70 | 51.45 | 39.24 |
| **Chr10** | 9.64 | 9.30 | 41.74 | 42.47 | 59.01 | 42.02 |
| **Chr11** | 8.98 | 7.21 | 41.19 | 40.09 | 54.03 | 43.79 |
| **Chr12** | 3.02 | 7.89 | 43.48 | 38.85 | 45.33 | 43.70 |
| **Chr13** | 41.94 | 6.12 | 54.85 | 39.87 | 31.61 | 42.52 |
| **Chr14** | - | 5.95 | - | 38.63 | - | 42.17 |
| **Chr15** | 26.66 | 7.79 | 43.01 | 39.95 | 48.86 | 44.27 |
| **Chr16** | 2.66 | 7.27 | 39.99 | 40.74 | 55.46 | 41.59 |
| **Chr17** | 9.38 | 9.43 | 37.02 | 41.58 | 61.69 | 42.07 |
| **Chr18** | 23.91 | 13.00 | 45.13 | 42.52 | 42.98 | 41.80 |
| **Chr19** | 4.60 | 3.57 | 39.92 | 38.24 | 55.44 | 44.41 |
| **Chr20** | - | 15.86 | - | 44.07 | - | 42.12 |
| **Chr21** | 6.17 | 9.59 | 43.19 | 39.99 | 48.92 | 46.19 |
| **Chr22** | - | 3.71 | - | 37.80 | - | 40.78 |
| **Chr23** | 23.17 | 6.29 | 39.56 | 39.75 | 36.75 | 44.88 |
| **Chr24** | - | 10.92 | - | 44.27 | - | 43.32 |
| **Chr25** | 12.58 | 7.70 | 41.57 | 41.25 | 47.11 | 42.89 |
| **Chr26** | 14.83 | 12.19 | 44.35 | 45.21 | 51.09 | 42.05 |
| **Chr27** | - | 11.55 | - | 39.96 | - | 44.54 |
| **Chr28** | - | 8.98 | - | 43.30 | - | 39.92 |
| **Chr29** | - | 4.61 | - | 38.02 | - | 44.44 |
| **Chr30** | 30.17 | 9.70 | 54.58 | 41.20 | 33.46 | 43.79 |
| **Chr31** | 6.83 | 5.89 | 44.29 | 39.36 | 59.24 | 40.47 |
| **Chr32** | - | 5.49 | - | 36.87 | - | 45.58 |
| **Chr33** | - | 7.17 | - | 38.95 | - | 42.98 |
| **Chr34** | - | 5.61 | - | 40.19 | - | 40.01 |
| **Chr35** | - | 8.52 | - | 41.16 | - | 42.72 |
| **Chr36** | - | 5.19 | - | 38.39 | - | 39.94 |
| **Chr37** | - | 7.31 | - | 39.91 | - | 39.32 |
| **Chr38** | - | 9.03 | - | 40.69 | - | 40.37 |
| **ChrX** | - | 6.67 | - | 40.06 | - | 56.84 |
| **Mean** | 17.07 | 8.37 | 44.12 | 40.79 | 49.01 | 42.85 |

**Table S15** Analysis of gene density, GC content and repetitive content for each chromosome in EBRs and whole genome in cat genome.

| **Chromosome** | **Mean gene density** | | **Percentage of GC content (%)** | | **Percentage of Repetitive content (%)** | |
| --- | --- | --- | --- | --- | --- | --- |
|  | **EBRs** | **Genome** | **EBRs** | **Genome** | **EBRs** | **Genome** |
| **A1** | 11.52 | 5.20 | 40.12 | 39.92 | 59.62 | 43.42 |
| **A2** | 5.68 | 9.26 | 43.08 | 42.29 | 40.76 | 42.54 |
| **A3** | 14.75 | 8.00 | 42.83 | 43.00 | 51.01 | 42.99 |
| **B1** | 6.37 | 4.72 | 39.24 | 39.18 | 48.30 | 45.60 |
| **B2** | 8.40 | 7.02 | 41.68 | 40.30 | 43.17 | 44.30 |
| **B3** | 13.88 | 7.99 | 39.91 | 41.96 | 45.37 | 44.95 |
| **B4** | 19.16 | 8.08 | 40.75 | 41.44 | 47.60 | 44.55 |
| **C1** | 12.16 | 8.09 | 43.93 | 41.59 | 43.59 | 43.82 |
| **C2** | 5.26 | 5.58 | 42.42 | 39.79 | 37.30 | 44.48 |
| **D1** | 13.56 | 11.36 | 42.47 | 42.66 | 42.89 | 44.91 |
| **D2** | 4.84 | 6.70 | 41.99 | 43.37 | 46.65 | 42.24 |
| **D3** | 18.48 | 7.30 | 46.58 | 43.35 | 40.93 | 41.63 |
| **D4** | 5.67 | 7.41 | 39.30 | 42.75 | 54.77 | 43.25 |
| **E1** | 7.31 | 16.39 | 50.70 | 47.21 | 50.14 | 40.40 |
| **E2** | 8.07 | 16.19 | 42.06 | 46.17 | 47.02 | 43.91 |
| **E3** | - | 12.41 | - | 48.34 | - | 43.00 |
| **F1** | 28.03 | 8.49 | 53.06 | 42.93 | 33.95 | 43.00 |
| **F2** | 0.00 | 4.04 | 37.83 | 40.74 | 54.07 | 43.99 |
| **X** | - | 5.28 | - | 40.63 | - | 57.41 |
| **Mean** | 10.77 | 8.40 | 42.82 | 42.51 | 46.30 | 44.23 |

**Table S16** Significantly enriched gene ontology (GO) biological process terms and KEGG pathways for the genes in EBRs of giant panda genome.

**Table S17** Significantly enriched gene ontology (GO) biological process terms and KEGG pathways for the genes in EBRs of dog genome.

**Table S18** Significantly enriched gene ontology (GO) biological process terms and KEGG pathways for the genes in EBRs of cat genome.
