# Supplementary material for: Chromosome-level genome assembly for giant panda provides novel insights into Carnivora chromosome evolution
Source: Genome Biol. 2019 Dec 6;20:267. doi: 10.1186/s13059-019-1889-7 (PMC6898958; doi:10.1186/s13059-019-1889-7)
Supplement: Supplementary file 3 — Additional file 3. Review history. [file 13059_2019_1889_MOESM3_ESM.docx]

Review History

**First round of review**

**Reviewer 1**

**Are you able to assess all statistics in the manuscript, including the appropriateness of statistical tests used?**

There are no statistics in the manuscript.

**Comments to author:**

This manuscript is about panda genome assembly at a chromosome level which was achieved by the usage of additional sequencing of 10x linked long

reads. It is an upgrade of an existing panda genome and the authors compared it with dog and cat chromosome assemblies.

It is a good resource to have such a high quality reference genome.

The authors did all the straightforward tasks to produce and compare it.

They found the obvious candidates of olfactory receptor related gene locations and also the pseudonization of the umami gene in the existing cat reference.

There are English errors (I guess they meant 'extensive' not 'expensive').

Overall, it is a good resource with default level comparative analyses.

This reviewer would accept this manuscript if the genome can be used extensively for future comparisons.

1. They did not cite the paper on carnivore genome compared with herbivore and omnivore

2. Is the new assembly from a different individual from the existing panda assembly(I.e., different blood samples)?

I guess the individual genetic diversity is significant.

3. "the flagship species" this is odd

4. "dramatically boosted the".. rephrase this.

5. "10X Genomics, as an alternative approach, which utilize genome partitioning and barcoding...." rephrase this.

6. "Moreover, our analyses demonstrated that 10X Genomic technology combined with flow-sorting and reference-assisted assembly allow for accurate construction of chromosome-level de novo assembly of large eukaryotic genomes.

==> this is well-established and not a great point to emphasize.

Essentially, the manuscript's value is about a new panda genome assembly with a long range information added and application of it for certain comparison.

The value or impact of the assembly is subjective.

7. "The results revealed that these three genomes had high genomic synteny. The pairwise synteny regions covered 2.30 Gb (95.0%) and 2.32 Gb (95.4%) of the giant panda and dog genomes

respectively;"

==> surprisingly well conserved. Interesting. What is the genomic distance denoted in time(years)?

8. EBR finding is useful and interesting.

The quality of PDF figures are not good somehow. They need to be improved.

**Reviewer 2**

**Are you able to assess all statistics in the manuscript, including the appropriateness of statistical tests used?**

There are no statistics in the manuscript.

**Comments to author:**

# Review of Fan et al MS on Giant panda chromosome -level genome assembly and analyses.

This manuscript is a detailed and thorough analyses of updated chromosome scale genome assembly , annotation and comparative analysis for the giant panda whole genome DNA sequence . This new study improves by a long shot the original Li et al 2010 offering on 2nd generation assembly of the panda .

The authors do a fine job in describing the weakness of previous assemblies in general and of the panda in particular by employing chromosome level assembly (long scaffolds), sequencing of flow sorted chromosomes to anchor the sequences to giant panda chromosomes by comparative identification previously demonstrated through chromosome paint studies, 10X Genomics Chromium and reference assisted assembly, and Illumina X Ten sequencing to generate paired end reads.

The starting material was fresh blood of a male panda at the Beijing Zoo.

The scaffolding and mapping were impressive showing 2.5 fold and 20-fold improvement in the scaffold N50 over previous giant panda assemblies.

I do offer a series of suggestions and a few concerns that once addressed will make a good study much stronger and clearer to the reader.

# ASSEMBLY

**1.** On page 7, the authors claim that the primary panda genome assembly was obtained from published paired-end and mate-pair reads and was extended using the 10X Genomics linked reads (lines 15–20). On page 19, the authors refer to the draft assembly, that seems to be the same as the primary assembly mentioned earlier, and to the Scaff10x program (lines 1–3). Does that mean that the linked reads by 10X Genomics were used only for scaffolding of scaffolds of the primary assembly? In other words, were the primary assembly contigs scaffolded two times: first using mate pairs and next using the linked reads? **Please clarify in text**

**2.** On page 9 (lines 12–16), the authors state that the giant panda scaffolds were linked and oriented using the cat genome as reference .However, on page 13 (lines 20–21) and page 19 (line 4) the dog genome is mentioned as reference for linking scaffolds. What genome was used as reference for arranging the assembled giant panda scaffolds on chromosomes: cat, dog, or both? **Please clarify in text.**

3. If the authors used the 10X Genomics linked reads only for scaffolding and did not perform their assembly, then genomic repeats of the giant panda genome might have been underrepresented in the assembly. That underrepresentation might have led to false conclusion about no significant difference for the

repeats in the cat genome EBRs (page 12, lines 11–12). **This should be stated in text.**

**4.** On page 20, the authors state that two panda chromosome pairs could not be resolved by the flow sorting: chromosomes 8 and X and chromosomes 10 and 11 (lines 3–4). Were scaffolds assigned to that chromosomes using evidence from the reference genome only? **Please clarify in text.**

5. Pairwise alignments is a rather outdated technique when talking about multiple species comparison. I couldn't actually find a good reason why one can not use multiple alignment for just a few species

**1.** (see Marguiles et al. "Differences between pair-wise and multi-sequence alignment methods affect vertebrate genome comparisons" (2006).

**2.** As one alternative, there is Progressive Mauve software that builds multiple genome alignment and directly produces synteny blocks from it. It should not demand too much resources just for three genomes. **Just a suggestion, not required if too much work.**

# Comparative EBR analysis for EBR identification.

The authors describe explicit syntenic segments ( also called **Homologous Synteny Blocks-HSBs**, an acronym they do not use), Chromosome Fissions, and Evolutionary Breakpoint Regions ( EBRs) between these three species .

They also use the genome segment alignments to map the panda HSBs to specific panda chromosomes from their sequencing of the flow sorted individual panda chromosomes. The mapping seems OK

However the comparative analysis (**page 10 line18 -page 12 line 3)needs a thorough reboot and rewrite .** This I believe is fixable but here are my concerns .

1.	On page 10 &14, they state *“Because the relative orientation of the giant panda scaffolds were unknown, we focused on inter****-****chromosome. rearrangement events in the present study*.” This means they annotate chromosome translocations only , and ignore inversions and transpositions within chromosomes in their comparisons.

a. This means we get the same analyses done before in several iterations with Chromosome Painting studies, several of which they do not cite. They claim that theirs is more fine tuned (it is) and their study finds:

*i.* *“We identified a total of 77 and 37 chromosome fission events. Compared with previous cytogenetic analysis, a total of 23 and 22 novel inter chromosome fission events were identified.”*

**b.** Nowhere do they actually compare their *inter chromosome fission* events to the ones we already know. **This explicit comparison should be added.**

c. There remains some confusion on my part. I am not sure what they mean by a Fission event (though I do know what a chromosome

fission is) and why it is so different from EBR. **Their count difference in fissions vs. EBRs are huge here.** Every breakpoint and rejoining someplace else produces an EBR and a rejoined fission that is also an EBR. So I am not sure what the point of using two terms and getting numbers that do not agree at all.

d. I suggest that in their Supplemental Tables they identify explicitly EBRs, and for the HSB, indicate Translocations, Transpositions within chromosomes, and inversions within chromosomes.

2.	The method used to define HSBs and EBRs is not so well defined. The paragraph on page 22 is not clear.

a. What are the anchors compared for syntenic orthology? Genes?, unique sequences, 5Mbs stretches , What?? . If they are genes, how precisely is homology confirmed for species that are 50MY apart? By the gene symbol? Not so good ! **Please explain better** .

b. How do you filter gene duplication, families, pseudogenes, and repeats in the definition of HSBs. This is hardly a routine exercise and the methods the authors used are not so clear to me. I cannot evaluate it very well.

c. Imprecise language in places makes me worry who wrote it

i. E.g. page 21 line 20 *“EBRs are adjacent syntenic fragments on the same reference chromosome.”* Huh???

3.	Please clearly describe how the authors built synteny blocks-HSBs, they do not mention neither software nor an algorithm that was used for this purpose. In fact, this is important, because many conclusions are built upon EBRs which are inferred directly from HSB junctions..

a. There are contradicting statements concerning reconstruction of HSBs . It's stated in Methods (p.20 line 17, p.21 line 18) that panda scaffolds were used for alignments and identification of HSBs among panda, dog and cat. However it contradicts Discussion (p.14, line 18):

i.	*"Our chromosome-level draft assembly of giant panda combined with published chromosome-level dog and cat genomes provide opportunities to identify novel chromosome rearrangements events among Carnivora that previous cytogenetic could not identify* ".

ii.	This sounds as if they used panda chromosomes instead of scaffolds for building synteny blocks. I believe it must become clear when (3) is fixed.

# 4. Minor things to be fixed

a. Page 4 line 11 I believe primitive carnivore is ringtail see

i.	Nash, W.G., Menninger, J.C., Padilla-Nash, H. M. and O’Brien, S.J.: Ancestral carnivore karyotype (2n=38) lives today in ringtails. J Hered 99: 241-253, 2008.

b. Figure resolution with PDF is terrible. I cannot read it

c. Page 8 line 15-18 is silly. Of course the whole genome include pairwise synteny segments They are called HSBs.

d. Fig 3 and the business about *TAS1R2 .* To me it seems presented as if it were something novel. It is not . It has been know for a decade- see citations below, none of which are cited. I might drop this discussion

;it adds little new information.

i.	Li X, Li W, Wang H, Bayley DL, Cao J, Reed DR, Bachmanov AA, Huang L,Legrand-Defretin V, Beauchamp GK, et al. 2006. Cats lack a sweet tastereceptor. J Nutr. 136:1932S–1934S.

ii.	Li X, Li W, Wang H, Cao J, Maehashi K, Huang L, Bachmanov AA, Reed DR, Legrand-Defretin V, Beauchamp GK, et al. 2005. Pseudogenization of a sweetreceptor gene accounts for cats’ indifference toward sugar. PLoSGenet. 1:27–35.

iii.	Li, X., Glaser, D., Li, W., Johnson, W.E., O'Brien, S.J., Beauchamp, G.K., Brand, J.G.: Analyses of sweet receptor gene (Tas1r2) and preference for sweet stimuli in species of Carnivora. J Hered. 2009 Jul-Aug;100 Suppl 1:S90-100.

e. Page 19line 14 ref 34 . Not sure this citation is correct. Actually, the authors should **check all the citations** because many of them are mixed (like [29] should be [28] for sure).

f. Citations for Chromosome painting of the three species should be checked and included :

g. More detail and citation is needed for transcriptome data when appropriate.

h. Did karyotype of cell line show any chromosome rearrangements during cell culture? **Say that**..

**i.** Regarding Conclusions , to me it sounds like a little bit of a stretch to claim that the findings in these three genomes characterize the whole Carnivora family. For examples the title of paragraph "Chromosomal rearrangements in Carnivora" (p.10, line 11), "Identification and analysis of EBRs in Carnivora" (p.11, line 3), conclusions about abundance of LINEs in Carnivora family (p.12 lines 5-7), and others. **Please clarify in text.**

In all, I liked the study and the paper a lot. Good Luck,

**Stephen J. O'Brien,**

Chief Scientific Officer

**Theodosius Dobzhansky Center**

**for Genome Bioinformatics**

St. Petersburg State University

**Authors Response**

**Point-by-point responses to the reviewers’ comments:**

Reviewer reports:

Reviewer #1: ===

This manuscript is about panda genome assembly at a chromosome level which was achieved by the usage of additional sequencing of 10x linked long reads. It is an upgrade of an existing panda genome and the authors compared it with dog and cat chromosome assemblies.

It is a good resource to have such a high quality reference genome. The authors did all the straightforward tasks to produce and compare it.

They found the obvious candidates of olfactory receptor related gene locations and also the pseudonization of the umami gene in the existing cat reference.

There are English errors (I guess they meant 'extensive' not 'expensive').

*Response: We have asked a native English-speaking researcher to help us polish the English of our revised manuscript.*

Overall, it is a good resource with default level comparative analyses.

This reviewer would accept this manuscript if the genome can be used extensively for future comparisons.

1. They did not cite the paper on carnivore genome compared with herbivore and omnivore

*Response: We have cited the paper (Kim et al, Genome biology, 2016) in the Introduction section of the revised manuscript (Lines 65-67).*

2. Is the new assembly from a different individual from the existing panda assembly (I.e., different blood samples)? I guess the individual genetic diversity is significant.

*Response: The previously published giant panda reference genomes of Li et al. (2010, Nature) and Hu et al. (2017, PNAS) were from the same individual (“Jingjing”, female).*

*In our study, the assembly process is as follows: the high-quality paired-end reads published by Hu et al. (2017) were used to assemble into contigs. Then these assembled contigs were scaffolded three times. Firstly, using the previously published mate-pair reads (Li et al., 2010), the contigs were merged into scaffolds with a scaffold N50 of 1.24 Mb. Then a blood sample from a different male giant panda was sequenced using 10X Genomics Chromium technology, and the linked reads were used to extend the primary scaffold assembly. Finally, based on the genome synteny results between giant panda and dog, we further extended the assembly and obtained a high-quality giant panda genome. Therefore, the new assembly is an improvement of the existing panda genome assembly, and the blood sample from a different male giant panda was just used for 10X Genomics library construction, sequencing and scaffolding. The SNP information of the 10X Genomics linked reads was not used in the process of genome assembly.*

3. "the flagship species" this is odd

*Response: We have changed ‘the’ to ‘a’ in the revised manuscript (Line 72).*

4. "dramatically boosted the".. rephrase this.

*Response: This sentence has been changed into “Recently, with the development of sequencing technologies, a combination of third generation sequencing techniques [18,19] and high-throughput chromatin conformation capture technique (Hi-C) [20] can produce high-quality genome assemblies, resulting in numerous chromosome-level draft genomes.” (Lines 100-104)*

5. "10X Genomics, as an alternative approach, which utilize genome partitioning and barcoding...." rephrase this.

*Response: This sentence has been changed into “The alternative approach of 10X Genomics employs genome partitioning and barcoding to generate linked reads that span tens to hundreds of thousands of bases [25]. These linked reads can be used to scaffold the contigs [26].” (Lines 110-113).*

6. "Moreover, our analyses demonstrated that 10X Genomic technology combined with flow-sorting and reference-assisted assembly allow for accurate construction of chromosome-level de novo assembly of large eukaryotic genomes.

==> this is well-established and not a great point to emphasize.

Essentially, the manuscript's value is about a new panda genome assembly with a long range information added and application of it for certain comparison.

The value or impact of the assembly is subjective.

*Response: This sentence has been deleted in the revised manuscript.*

7. "The results revealed that these three genomes had high genomic synteny. The pairwise synteny regions covered 2.30 Gb (95.0%) and 2.32 Gb (95.4%) of the giant panda and dog genomes

respectively;"

==> surprisingly well conserved. Interesting. What is the genomic distance denoted in time (years)?

*Response: To investigate Carnivora genome conservation, we performed multiple genome alignment among the three genomes using Progressive Mauve software. The results showed that the total sizes of syntenic regions among the giant panda scaffolds, the dog and cat chromosomes were 2.29 Gb (93.70% of the genome), 2.27 Gb (93.10% of the genome) and 2.35 Gb (96.19% of the genome), respectively. The comparative results between the giant panda and dog genomes obtained in this study were similar to previously published results (Li et al., 2010), which showed that the total sizes of syntenic regions between the giant panda and dog genomes were 2.22 Gb (96.7% of the genome) and 2.27 Gb (92.9% of the genome), respectively.*

*According to the Timetree (http://www.timetree.org/) database, the genomic distance between giant panda and dog is 46 MYA, and the genomic distance between giant panda and cat is 54 MYA. The high level of synteny among these three genomes was not surprising despite the long genomic distance. There are many similar examples. For example, the synteny regions between mouse and cat genome, which have a genomic distance of 96 MYA, represent 92.41% of the mouse genome; the synteny regions between mouse and horse gnome, which have a genome distance of 96 MYA, represent 93.49% of the mouse genome (Capilla et al., 2016).*

8. EBR finding is useful and interesting.

*Response: Thanks for your comment.*

The quality of PDF figures are not good somehow. They need to be improved.

*Response: We have provided high-quality figures in the revised manuscript.*

Reviewer #2: ===

This manuscript is a detailed and thorough analyses of updated chromosome scale genome assembly, annotation and comparative analysis for the giant panda whole genome DNA sequence. This new study improves by a long shot the original Li et al 2010 offering on 2nd generation assembly of the panda.

The authors do a fine job in describing the weakness of previous assemblies in general and of the panda in particular by employing chromosome level assembly (long scaffolds), sequencing of flow sorted chromosomes to anchor the sequences to giant panda chromosomes by comparative identification previously demonstrated through chromosome paint studies, 10X Genomics Chromium and reference assisted assembly, and Illumina X Ten sequencing to generate paired end reads. The starting material was fresh blood of a male panda at the Beijing Zoo. The scaffolding and mapping were impressive showing 2.5 fold and 20-fold improvement in the scaffold N50 over previous giant panda assemblies. I do offer a series of suggestions and a few concerns that once addressed will make a good study much stronger and clearer to the reader.

*Response: Thanks for your comments.*

ASSEMBLY

1. On page 7, the authors claim that the primary panda genome assembly was obtained from published paired-end and mate-pair reads and was extended using the 10X Genomics linked reads (lines 15–20). On page 19, the authors refer to the draft assembly, that seems to be the same as the primary assembly mentioned earlier, and to the Scaff10x program (lines 1–3). Does that mean that the linked reads by 10X Genomics were used only for scaffolding of scaffolds of the primary assembly? In other words, were the primary assembly contigs scaffolded two times: first using mate pairs and next using the linked reads? Please clarify in text

*Response: Thanks for the comment. Yes, the primary genome assembly of giant panda was scaffolded three times. Based on the previously published paired-end reads generated with Illumina sequencing platforms (Hu et al., 2017), the giant panda genome was assembled into contigs. Then the primary contigs were scaffolded three times. First, using previously published mate-pair reads (Li et al., 2010), the contigs were merged into scaffolds with N50 of 1.24 Mb. Second, a male giant panda was sequenced using 10X Genomics Chromium technology, and a total of 250 Gb linked reads were generated (104.2-fold genome coverage). Using these 10X Genomics linked reads, we extended the primary assembly to an assembly with a scaffold N50 of 18.03 Mb Finally, based on the genome synteny results between giant panda and dog, we further extended the assembly and obtained a high-quality giant panda genome with a total size of 2.45 Gb and a scaffold N50 of 23.47 Mb (Table S1). (Lines 136-146).*

2. On page 9 (lines 12–16), the authors state that the giant panda scaffolds were linked and oriented using the cat genome as reference. However, on page 13 (lines 20–21) and page 19 (line 4) the dog genome is mentioned as reference for linking scaffolds. What genome was used as reference for arranging the assembled giant panda scaffolds on chromosomes: cat, dog, or both? Please clarify in text.

*Response: Thank you for the good comment. In fact, the cat genome was used as reference for arranging the assembled giant panda scaffolds on the chromosomes. The dog genome was just used to extend the scaffolds of the giant panda genome. We have clarified it in the Materials and Methods section of the revised manuscript (Lines 404-406, Lines 444-451).*

3. If the authors used the 10X Genomics linked reads only for scaffolding and did not perform their assembly, then genomic repeats of the giant panda genome might have been underrepresented in the assembly. That underrepresentation might have led to false conclusion about no significant difference for the repeats in the cat genome EBRs (page 12, lines 11–12). This should be stated in text.

*Response: Thanks for your comments. We agree with that because the 10X Genomics linked reads was not used for de novo assembly, the repeat sequences of the giant panda genome might be underrepresented in the assembly. However, in our study, the comparisons between the EBR and the whole genome were performed for giant panda, dog, and cat, respectively. So, our understanding is that the comparison of the repeat sequences between the EBR and the whole genome for cat was not affected by the giant panda genome assembly, because we have reduced the number of raw local synteny blocks resulting from the repeat sequences in the identification of HSBs for three Carnivora genomes. On the other hand, the reason that we did not perform de novo assembly using 10X Genomics linked reads was that the 10X sequenced individual is a male giant panda, different from the original sequenced female giant panda “Jingjing”. We want to obtain a genome assembly from a single giant panda, rather than the mixed individuals. We have added this related statement in the Materials and Methods section part of the revised manuscript. (Lines 402-404)*

4. On page 20, the authors state that two panda chromosome pairs could not be resolved by the flow sorting: chromosomes 8 and X and chromosomes 10 and 11 (lines 3–4). Were scaffolds assigned to that chromosomes using evidence from the reference genome only? Please clarify in text.

*Response: Thanks for your comments. We are sorry for the numbering mistake for the chromosomes 8 and 9. Actually, the chromosome 9 was not separated from X. Two pairs of chromosomes, chromosomes 9 and X, chromosomes 10 and 11, could not be resolved by flow-sorting analysis. However, they can be discriminated into an individual chromosome using the evidence from the cat genome. We have clarified this in the Results section of the revised manuscript (Lines 189-191).*

5. Pairwise alignments is a rather outdated technique when talking about multiple species comparison. I couldn't actually find a good reason why one can not use multiple alignment for just a few species 1. (see Marguiles et al. "Differences between pair-wise and multi-sequence alignment methods affect vertebrate genome comparisons" (2006).

2. As one alternative, there is Progressive Mauve software that builds multiple genome alignment and directly produces synteny blocks from it. It should not demand too much resources just for three genomes. Just a suggestion, not required if too much work.

*Response: Thanks for your good comments. Following your suggestion, we have aligned the scaffolds of giant panda to dog and cat chromosomes using Progressive Mauve software. The results showed that the total size of synteny regions among giant panda, dog and cat genomes represented 2.29 Gb (93.70%), 2.27 Gb (93.10%) and 2.35 Gb (96.19%) of giant panda, dog and cat genome, respectively.*

However, previous studies revealed that Mauve software could not identify large-scale synteny blocks, but rather smaller-scale ‘locally collinear blocks’ because this software was developed to perform comparative analysis of bacterial genomes (Soderlund et al., 2011). In this study, we focused on the chromosome fission events and EBRs among three large-size Carnivora genomes. Thus, the small-scale synteny blocks were not suitable for this study. Therefore, to identify large-scale HSBs, we used the pairwise alignment strategy SyMAP to align the giant panda chromosomes to dog and cat chromosomes.

Comparative EBR analysis for EBR identification. The authors describe explicit syntenic segments (also called Homologous Synteny Blocks-HSBs, an acronym they do not use), Chromosome Fissions, and Evolutionary Breakpoint Regions (EBRs) between these three species. They also use the genome segment alignments to map the panda HSBs to specific panda chromosomes from their sequencing of the flow sorted individual panda chromosomes. The mapping seems OK

*Response: Thanks for your comments.*

However, the comparative analysis (page 10 line18 -page 12 line 3) needs a thorough reboot and rewrite. This I believe is fixable but here are my concerns.

1. On page 10 &14, they state “Because the relative orientation of the giant panda scaffolds were unknown, we focused on inter-chromosome. rearrangement events in the present study.” This means they annotate chromosome translocations only, and ignore inversions and transpositions within chromosomes in their comparisons.

a. This means we get the same analyses done before in several iterations with Chromosome Painting studies, several of which they do not cite. They claim that theirs is more fine tuned (it is) and their study finds: i. “We identified a total of 77 and 37 chromosome fission events. Compared with previous cytogenetic analysis, a total of 23 and 22 novel inter chromosome fission events were identified.”

b. Nowhere do they actually compare their inter chromosome fission events to the ones we already know. This explicit comparison should be added

*Response: Thanks for your comments. We have added the detailed comparisons between chromosome fission events detected in this study and in other FISH-based cytogenetic studies in the Table S3. The description about the comparison has been added in the Results (Lines 212-217) and Discussion (Lines 309-317) sections of revised manuscripts.*

c. There remains some confusion on my part. I am not sure what they mean by a Fission event (though I do know what a chromosome 3 fission is) and why it is so different from EBR. Their count difference in fissions vs. EBRs are huge here. Every breakpoint and rejoining someplace else produces an EBR and a rejoined fission that is also an EBR. So I am not sure what the point of using two terms and getting numbers that do not agree at all.

*Response: Thanks for your comments. We are sorry for the confusion due to the mistakes. As you commented, in this study, the number of chromosome fission events was similar to that of the EBRs. For instance, a total of 59 and 16 chromosome fission events were identified when we aligned the dog and cat genomes to the giant panda chromosomes. Whereas the alignment of the dog and cat genomes to the giant panda chromosomes revealed a total of 58 and 15 EBRs, 14 of which presented overlapped regions in the giant panda genome. We have corrected this in the revised manuscript (Lines 212-214, Lines 230-232).*

d. I suggest that in their Supplemental Tables they identify explicitly EBRs, and for the HSB, indicate Translocations, Transpositions within chromosomes, and inversions within chromosomes.

*Response: Thanks for your comments. We have added the information of HSBs in Table S4-S6 in the revised manuscripts, including the translocations, inversion within chromosomes, and transposition within chromosomes.*

2. The method used to define HSBs and EBRs is not so well defined. The paragraph on page 22 is not clear.

a. What are the anchors compared for syntenic orthology? Genes?, unique sequences, 5Mbs stretches , What?? . If they are genes, how precisely is homology confirmed for species that are 50MY apart? By the gene symbol? Not so good ! Please explain better .

*Response: Thanks for your good comments. We manually built the HSBs in our original manuscript. Following your suggestion, we used SyMAP software to build large-scale HSBs. The SyMAP program firstly aligned the genomic sequence using MUMmer method to form raw local synteny blocks which were defined as 20 base-pair or longer exact matches between two genomes. Then the raw local synteny blocks were clustered and filtered using the gene annotation to form anchors. We have clarified it in the Materials and Methods section of the revised manuscript. (Lines 477-486)*

b. How do you filter gene duplication, families, pseudogenes, and repeats in the definition of HSBs. This is hardly a routine exercise and the methods the authors used are not so clear to me. I cannot evaluate it very well.

*Response: The OrthoFinder software was used to detect orthologous genes, and the orthologous protein-coding genes among the three genomes were used as the input file for SyMAP. So, the analysis did not use the gene duplication and gene families. The pseudogenes were filtered out in the orthologous gene analysis. The SyMAP reduces the number of raw local synteny blocks resulting from the repeat sequences with several stages of clustering and filtering based on orthologous genes. First, the existing set of annotated genes (AGs) for each genome is augmented with un-annotated clusters (UCs), which are created from the MUMmer local synteny blocks that do not overlap gene annotation on that genome. Second, the MUMmer local synteny blocks were grouped into ‘anchors’ using the AGs and UCs. Finally, the anchors are filtered using the criterion that an anchor must be one of the top 2 anchor scores for both its query and target side.*

c. Imprecise language in places makes me worry who wrote it i. E.g. page 21 line 20 “EBRs are adjacent syntenic fragments on the same reference chromosome.” Huh???

*Response: We are sorry for this mistake. This sentence has been changed to “The EBRs are defined as the interval between two large-scale HSBs that is demarcated by the end-sequence coordinates of large-scale HSBs on each side.” (Lines 487-489).*

3. Please clearly describe how the authors built synteny blocks-HSBs, they do not mention neither software nor an algorithm that was used for this purpose. In fact, this is important, because many conclusions are built upon EBRs which are inferred directly from HSB junctions.

*Response: Thanks for your good comments. In fact, we manually built the HSBs in our original manuscript. Following your suggestion, we used the software SyMAP (Synteny mapping and analysis program) to build large-scale HSBs in the revised manuscript. Briefly, the SyMap program firstly aligned the genomic sequence using MUMmer method [53]. Then the raw local synteny blocks were clustered and filtered using the gene annotation to form anchors. The anchors were input into the synteny algorithm, which was designed to form large-scale HSBs, where intervening micro-rearrangements were allowed. We have clarified this in the Materials and Methods section of the revised manuscripts (Lines 476-485).*

a. There are contradicting statements concerning reconstruction of HSBs. It's stated in Methods (p.20 line 17, p.21 line 18) that panda scaffolds were used for alignments and identification of HSBs among panda, dog and cat. However it contradicts Discussion (p.14, line 18):

i. "Our chromosome-level draft assembly of giant panda combined with published chromosome-level dog and cat genomes provide opportunities to identify novel chromosome rearrangements events among Carnivora that previous cytogenetic could not identify ".

ii. This sounds as if they used panda chromosomes instead of scaffolds for building synteny blocks. I believe it must become clear when (3) is fixed.

*Response: In fact, the giant panda chromosomes were used to detect synteny blocks, whereas the giant panda scaffolds were aligned to the cat and dog chromosomes to verify that the sequences of giant panda, dog and cat genomes are largely conserved.*

4. Minor things to be fixed

a. Page 4 line 11 I believe primitive carnivore is ringtail see i. Nash, W.G., Menninger, J.C., Padilla-Nash, H. M. and O’Brien, S.J.: Ancestral carnivore karyotype (2n=38) lives today in ringtails. J Hered 99: 241-253, 2008.

*Response: We have changed the sentence as “The karyotypes of the giant panda (2n = 42) and cat (2n = 38) are similar to that of ancestral Carnivora which lives today in ringtails (2n = 38)” and added this reference in the revised manuscript (Line 53).*

b. Figure resolution with PDF is terrible. I cannot read it

*Response: We have provided high-quality figures in the revised manuscript.*

c. Page 8 line 15-18 is silly. Of course the whole genome include pairwise synteny segments They are called HSBs.

*Response: Thank you for your comments. We have changed this sentence as “The results showed that the total size of synteny regions among giant panda scaffolds, dog and cat chromosomes represented 2.29 Gb (93.70%), 2.27 Gb (93.10%) and 2.35 Gb (96.19%), respectively.” (Line 160-163).*

d. Fig 3 and the business about TAS1R2 . To me it seems presented as if it were something novel. It is not. It has been know for a decade- see citations below, none of which are cited. I might drop this discussion;it adds little new information.

i. Li X, Li W, Wang H, Bayley DL, Cao J, Reed DR, Bachmanov AA,

Huang L,Legrand-Defretin V, Beauchamp GK, et al. 2006. Cats lack a sweet tastereceptor. J Nutr. 136:1932S–1934S.

ii. Li X, Li W, Wang H, Cao J, Maehashi K, Huang L, Bachmanov AA, Reed DR, Legrand-Defretin V, Beauchamp GK, et al. 2005.

Pseudogenization of a sweetreceptor gene accounts for cats’ indifference toward sugar. PLoSGenet. 1:27–35.

iii. Li, X., Glaser, D., Li, W., Johnson, W.E., O'Brien, S.J., Beauchamp, G.K., Brand, J.G.: Analyses of sweet receptor gene (Tas1r2) and preference for sweet stimuli in species of Carnivora. J Hered. 2009 Jul-Aug;100 Suppl 1:S90-100.

*Response: Thank you for your comments. We have added the citations in the revised manuscript (Line 268). Because the gene TAS1R2 was identified to be located in the EBR region of giant panda, the discussion about TAS1R2 was to explain that the chromosome rearrangement may play a role in the pseudogenization of TAS1R2 in cat.*

e. Page 19line 14 ref 34 . Not sure this citation is correct. Actually, the authors should check all the citations because many of them are mixed (like [29] should be [28] for sure).

*Response: We have removed the ref 34 and checked all of the references cited in this manuscript.*

f. Citations for Chromosome painting of the three species should be checked and included :

*Response: We have added all of the citations for chromosome painting of three species in the revised manuscript (Line 57).*

g. More detail and citation is needed for transcriptome data when appropriate.

*Response: These transcript sequences were assembled using RNA-seq data for 15 samples from five giant pandas (two blood samples from two females, one blood sample from one male, pallium, liver, small intestine, stomach, colon, and testis samples from one male adult; skeletal muscle, pituitary, tongue, ovary, and two skin tissue samples from one female adult). We have added it in the revised manuscript (Lines 466-470).*

h. Did karyotype of cell line show any chromosome rearrangements during cell culture? Say that..

*Response: We did not find any chromosome rearrangements when we performed the flow sorting.*

i. Regarding Conclusions, to me it sounds like a little bit of a stretch to claim that the findings in these three genomes characterize the whole Carnivora family. For examples the title of paragraph "Chromosomal rearrangements in Carnivora" (p.10, line 11), "Identification and analysis of EBRs in Carnivora" (p.11, line 3), conclusions about abundance of LINEs in Carnivora family (p.12 lines 5-7), and others. Please clarify in text.

*Response: Thanks for your comments. We have removed these expressions in the revised manuscript.*

*Reference*

*1. Kim S, Cho YS, Kim HM, Chung O, Kim H, et al. Comparison of carnivore, omnivore, and herbivore mammalian genomes with a new leopard assembly. Genome Biol. 2016; 17:211.*

*2. Li R, Fan W, Tian G, Zhu H, He L, Cai J, et al. The sequence and de novo assembly of the giant panda genome. Nature. 2010;463:311-7.*

*3. Hu Y, Wu Q, Ma S, Ma T, Shan L, Wang X, et al. Comparative genomics reveals convergent evolution between the bamboo-eating giant and red pandas. Proc Natl Acad Sci U S A. 2017;114:1081-6.*

*4. Capilla L, Sanchez-Guillen RA, Farre M, Paytuvi-Gallart A, Malinverni R, Ventura J, Larkin DM, Ruiz-Herrera A: Mammalian Comparative Genomics Reveals Genetic and Epigenetic Features Associated with Genome Reshuffling in Rodentia. Genome Biol Evol 2016, 8:3703-3717.*

*5. Nie W, Wang J, Su W, Wang D, Tanomtong A, Perelman PL, et al. Chromosomal rearrangements and karyotype evolution in carnivores revealed by chromosome painting. Heredity. 2012;108:17-27.*

*6. Soderlund C, Bomhoff M, Nelson WM. SyMAP v3.4: a turnkey synteny system with application to plant genomes. Nucleic Acids Res. 2011; 39:e68.*

**Second round of review**

**Reviewer 2**

line 59 ref 2 not 3

line 268 mention here that cats lose sweetness taste as a phenotypic consequence of the pseudogenization of TAS1R2 ( dogs do not)

line 331 probably should cite their ref 3 and also

O’Brien, S.J., Eisenberg, J.F., Miyamoto, M., Hedges, S.B., Kumar, S., Wilson, D.E. Minotti-Raymond, M., Murphy, W.J., Nash, W.G., Lyons, L.A., Menninger, J.C., Stanyon, R., Wienberg, J., Copeland, N.G., Jenkins, N.A., Gellin, J., Yerle, J., Andersson, L., Womack, J., Broad, T., Postlethwait, J., Serov, O., Bailey E., James, M.R., Marshal Graves, J.A., Genone Maps 10. Comparative genomics. Mammalian radiations. Wall chart. Science 286:463-478,1999.

**Authors Response**

**Point-by-point responses to the reviewers’ comments:**

Reviewer #2:

1. line 59 ref 2 not 3

*Response: We have changed the reference here.*

2. line 268 mention here that cats lose sweetness taste as a phenotypic consequence of the pseudogenization of TAS1R2 ( dogs do not)

*Response: We have added the sentence “The pseudogenization of TAS1R2 may make cat insensitive to sweet-tasting compounds compared to dog’s normal sweetness taste” here in the revised manuscript.*

3. line 331 probably should cite their ref 3 and also

O’Brien, S.J., Eisenberg, J.F., Miyamoto, M., Hedges, S.B., Kumar, S., Wilson, D.E. Minotti-Raymond, M., Murphy, W.J., Nash, W.G., Lyons, L.A., Menninger, J.C., Stanyon, R., Wienberg, J., Copeland, N.G., Jenkins, N.A., Gellin, J., Yerle, J., Andersson, L., Womack, J., Broad, T., Postlethwait, J., Serov, O., Bailey E., James, M.R., Marshal Graves, J.A., Genone Maps 10. Comparative genomics. Mammalian radiations. Wall chart. Science 286:463-478,1999.

*Response: We have added the reference 3 and the citation in the revised manuscript.*
